# Combined Mitochondrial and Nuclear Markers Revealed a Deep Vicariant History for *Leopoldamys neilli*, a Cave-Dwelling Rodent of Thailand

**DOI:** 10.1371/journal.pone.0047670

**Published:** 2012-10-31

**Authors:** Alice Latinne, Surachit Waengsothorn, Prateep Rojanadilok, Krairat Eiamampai, Kriangsak Sribuarod, Johan R. Michaux

**Affiliations:** 1 Laboratoire de génétique des microorganismes, Institut de Botanique, University of Liège, Liège, Belgium; 2 Environment and Resources Technology Department, Thailand Institute of Scientific and Technological Research, Pathum Thani, Thailand; 3 Doi Chiang Dao Wildlife Research Station, Chiang Mai, Thailand; 4 Bung Boraphet Wildlife Research Station, Nakhon Sawan, Thailand; 5 Khlong Saeng Wildlife Research Station, Surat Thani, Thailand; 6 INRA, UMR CBGP (INRA/IRD/Cirad/Montpellier SupAgro), Campus International de Baillarguet, Montferrier-sur-Lez, France; University of Otago, New Zealand

## Abstract

**Background:**

Historical biogeography and evolutionary processes of cave taxa have been widely studied in temperate regions. However, Southeast Asian cave ecosystems remain largely unexplored despite their high scientific interest. Here we studied the phylogeography of *Leopoldamys neilli*, a cave-dwelling murine rodent living in limestone karsts of Thailand, and compared the molecular signature of mitochondrial and nuclear markers.

**Methodology/Principal Findings:**

We used a large sampling (n = 225) from 28 localities in Thailand and a combination of mitochondrial and nuclear markers with various evolutionary rates (two intronic regions and 12 microsatellites). The evolutionary history of *L. neilli* and the relative role of vicariance and dispersal were investigated using ancestral range reconstruction analysis and Approximate Bayesian computation (ABC).

Both mitochondrial and nuclear markers support a large-scale population structure of four main groups (west, centre, north and northeast) and a strong finer structure within each of these groups. A deep genealogical divergence among geographically close lineages is observed and denotes a high population fragmentation. Our findings suggest that the current phylogeographic pattern of this species results from the fragmentation of a widespread ancestral population and that vicariance has played a significant role in the evolutionary history of *L. neilli*. These deep vicariant events that occurred during Plio-Pleistocene are related to the formation of the Central Plain of Thailand. Consequently, the western, central, northern and northeastern groups of populations were historically isolated and should be considered as four distinct Evolutionarily Significant Units (ESUs).

**Conclusions/Significance:**

Our study confirms the benefit of using several independent genetic markers to obtain a comprehensive and reliable picture of *L. neilli* evolutionary history at different levels of resolution. The complex genetic structure of *Leopoldamys neilli* is supported by congruent mitochondrial and nuclear markers and has been influenced by the geological history of Thailand during Plio-Pleistocene.

## Introduction

Subterranean habitats and their singular fauna have fascinated biologists and biogeographers for centuries. Historically, there has been a long debate over the hypotheses regarding the evolution of subterranean animals and particularly the relative role of vicariance and dispersal and the influence of potential isolating barriers to explain biogeographic patterns of these taxa [1], [2]. According to the vicariant hypothesis, the ancient range expansion of a large ancestral population is followed by its fragmentation due to the appearance of isolating barrier [3]–[5]. In contrast, according to the dispersalist hypothesis, active colonization of new habitats from a small centre of origin happens across pre-existing barriers [2], [5]. It is now suggested that a combination of both vicariance and dispersal better explains the biogeography of several subterranean taxa rather than a single model (see [1], [2], [6] for a review).

The modes of speciation of subterranean animals have also been debated and several models proposed: the Climatic Relict Hypothesis (CRH) [7] and the Adaptive Shift Hypothesis (ASH) [8], [9]. The CRH was suggested for temperate ecosystems where unfavourable environmental conditions during Pleistocene climatic fluctuations divided an ancestral surface population into subterranean populations which retreated beneath the surface, and surface populations which may have become extinct or have migrated. To explain the evolution of subterranean fauna in the tropics, an alternative model, the ASH, was proposed. This model assumes an active colonization of subsurface habitats to exploit new resources coupled with an adaptive differentiation of surface and subterranean populations and possible gene flow between them for some time before genetic isolation and parapatric speciation occurred. Support for both models has been obtained for diverse species in different regions of the world using phylogenetic analysis, molecular dating and analysis of the current geographical distributions of taxa [10]–[12].

Due to their discontinuous distribution and the peculiarity of their fauna, limestone karsts constitute an interesting model to explore these evolutionary processes. Limestone karsts are sedimentary rock outcrops consisting of calcium carbonate sculpted by solutional erosion into the typical landscape made of cone-shaped or sheer-sided hills riddled with caves and sinkholes [13]. Karst biodiversity is characterized by high levels of endemic species adapted to this extreme environment and include a wide variety of organisms among which some species are permanent subterranean resident in caves while others are associated with karst surface [14]. Most of these species are rare and confined to small geographic areas [13], [14]. Studies of karst and cave organisms available in the literature have mainly focused on temperate ecosystems [10], [13] while karst biodiversity remain largely unexplored in Southeast Asia, even though this region encompassing four biodiversity hotspots [15] has some of the largest limestone karst regions of the world which cover an area of 460000 km^2^ [16].

In order to improve our knowledge of karst biodiversity in southeast asian limestone karsts and to better understand the biogeographic processes operating in these habitats, we studied the phylogeography and evolutionary history of Neill's rat *Leopoldamys neilli* (Marshall, 1976), a murine rodent species endemic to limestone karsts of Thailand, within the Indo-Burmese biodiversity hotspot. The species was discovered in karsts of the Saraburi province, central Thailand and has also been recorded in few locations in northern and western Thailand (Figure S1) but the limits of its geographical range are not clearly resolved [17]–[20]. Following the classification of Sket [21], *L. neilli* is a subtroglophilic species, i.e., a resident of subterranean habitat associated with karst surface to complete some parts of its life cycle [22]. This species was listed as “Endangered” on the IUCN Red List but is now classified as “Data deficient” due to the lack of data about its distribution and ecological requirements [23]. Two additional *Leopoldamys* species, *L. edwardsi* and *L. sabanus*, both semi-arboreal rodents living in forest ecosystems, also occur in Thailand. A recent molecular phylogeny suggested that *L. neilli* and *L. edwardsi* are sister species [24].

A preliminary study of the phylogeography of *L. neilli* based on one nuclear and two mitochondrial fragments revealed a strong geographic structure of the mitochondrial DNA (mtDNA) genetic diversity for this species [25]: six highly divergent and allopatric genetic lineages that were isolated for a long time were observed in Thailand. However, the nuclear fragment used in this study (the intron 7 of the β-fibrinogen gene) showed a very weak genetic variability within the study area and presented no phylogeographic signal. Therefore conclusions of this previous study were mainly derived from mtDNA. Due to its numerous advantages such as high rate of evolution, lack of recombination and haploidy, mtDNA has been widely used as a classical phylogeographic marker. However, due to its maternal inheritance and risks of introgression, mtDNA also presents some inconveniences and could yield to biased phylogenies that are not representative of the actual species tree (e.g. [26]–[28]). Therefore, to avoid this trouble and to refine the rather conflicting results obtained from a single nuclear fragment and two mitochondrial markers in Latinne *et al.* [25], the combined analysis of multiple independent loci with various evolutionary rates was required to corroborate or reject the hypotheses previously established in this last study. Moreover, the sample size of this previous study was excessively small for some regions of northern Thailand and needed to be expanded with more samples and more localities to yield reliable conclusions.

Using a much larger sampling and various nuclear markers, the present study was conducted to address the following questions: (i) How are *L. neilli* populations genetically structured throughout Thailand according to mitochondrial and nuclear markers? (ii) Is the phylogeograhic structure of mtDNA representative of the evolutionary history of *L. neilli*? Are molecular signatures of mitochondrial and nuclear markers congruent? (iii) When did the divergence between *L. neilli* and *L. edwardsi* occur and what can we infer about the speciation process of *L. neilli*? (iv) Where were *L. neilli* ancestors distributed? What are the relative roles of vicariance, dispersal and potential isolating barriers in the evolutionary history of *L. neilli*? (v) According to its distribution and genetic structure, which conservation status should be given to this species?

## Materials and Methods

### Ethics statement

Sample collection was carried out in collaboration with researchers located at the Thailand Institute of Scientific and Technological Research (TISTR) and directors of Wildlife Research Stations (under the administration of the Department of National Park, Wildlife and Plant Conservation in Thailand) having the required permits. This study is part of the “CERoPath project” (Community Ecology of Rodents and their Pathogens in South-East Asia: effects of biodiversity changes and implications in health ecology), ANR Biodiversity ANR07 BDIV012. CERoPath protocols (available at http://www.ceropath.org/references/rodent_protocols_book) were evaluated by the ethics committee of Mahidol University in Bangkok.

### Sampling and DNA extraction

**Figure 1:**
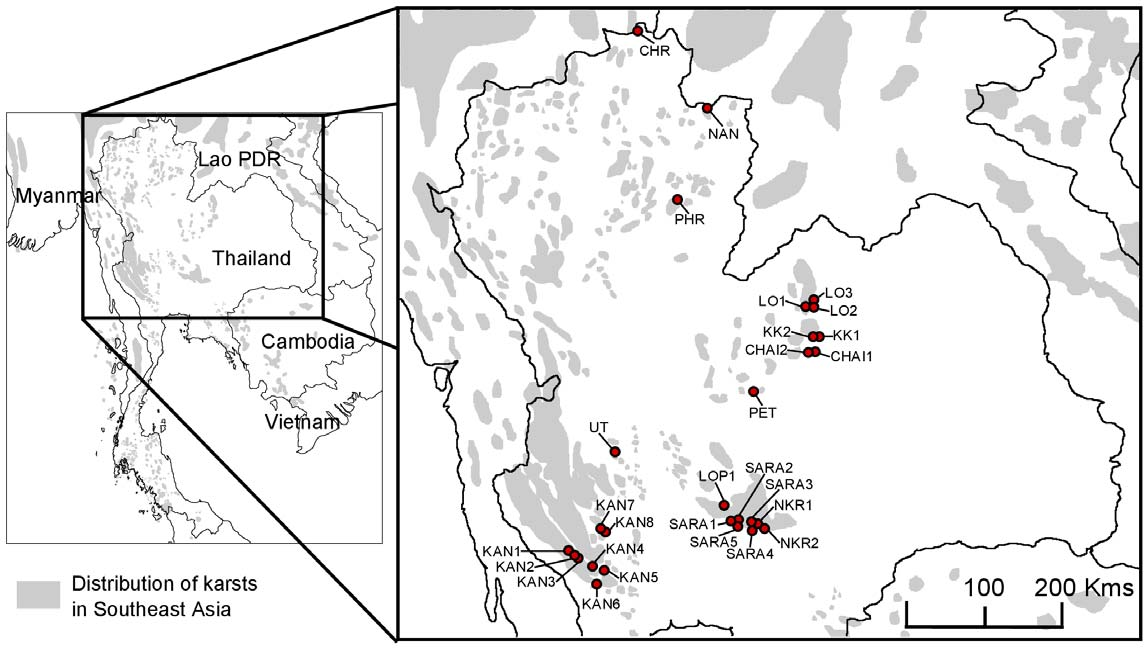
Sampling localities of the 225 *L. neilli* analyzed in this study.

A total of 225 samples of *L. neilli* collected in 28 localities (Figure 1, Table S1) during a two-year survey of 122 limestone karsts distributed in all karstic regions of Thailand were used in this study (Figure S1). These 225 samples include 115 specimens from a previous study [25] and 110 new individuals, among which 50 come from eight localities (CHAI1, CHAI2, CHR, KK1, KK2, PET, SARA5, UT) unsampled in Latinne *et al.* [25]. Field identifications were made based on morphological criteria according to [17], [29]. Animals were live-trapped and released after taking a tissue biopsy from the ear. Skin samples were stored in 96% ethanol. Genomic DNA was extracted from skin samples using the DNeasy Tissue Kit (Qiagen Inc.) following the manufacturer's protocol.

For phylogenetic analysis, six samples of *L. edwardsi* and *L. sabanus* (5 voucher specimens from the CERoPath tissue collection (R3033 (HM217400, HM217531, HQ454288, JQ081354), R3111 (HM217404, HM217534), R4222 (HM217444, HM217571, HQ454297, JQ081352), R4296 (HM217450, HM217577, HQ454292, JQ081353), R4370 (HM217451, HM217578, HQ454293, JQ081351)) [24] and one specimen collected by our team (L288 (JQ081371, JQ081319, JQ081342, JQ081355)) were chosen as outgroups (Table S2).

### Mitochondrial and nuclear DNA amplification

Two mitochondrial markers, the cytochrome *b* gene (cytb) and the cytochrome oxidase I gene (COI), were used in this study in addition to two nuclear fragments; the intron 7 region of the β-fibrinogen gene (bfibr) and the intron 1 region of the X-linked glucose-6-phosphate dehydrogenase gene (G6pd). Sequences of cytb (n = 115), COI (n = 115) and bfibr (n = 65) were available from a previous study [25] and were associated to newly amplified sequences. The final dataset included 225 sequences of mitochondrial genes (cytb, COI) and 104 sequences of nuclear introns (bfibr, G6PD) from a subset of 104 individuals representative of the main sampling localities. Primers sets used to amplify the cytb, COI, bfibr and G6pd genes and their annealing temperatures are listed in Table S3. PCRs were carried out in 50 µl volume containing 12.5 µl of each 2 µM primers, 1 µl of 10 mM dNTP, 10 µl of 5× reaction buffer (Promega), 0.2 µl of 5 U/µl Promega Taq DNA Polymerase and approximately 30 ng of DNA extract. Amplifications were performed in thermal cycler VWR Unocycler using one activation step (94°C for 4 min) followed by 40 cycles (94°C for 30 sec, 50–58.7°C (depending on the primers, Table S3) for 60 sec, and finally 72°C for 90 sec) with a final extension step at 72°C for 10 min. Sequencing reactions were performed by Macrogen Inc. (Seoul, Korea) using sequencing on an ABI 3730 automatic sequencer.

All haplotype/allele sequences have been deposited in Genbank database (GenBank Accession Numbers: cytb: HM219591–612, JQ081356–370; COI: HM219573–588, JQ081310–318; bfibr: HM219556–560, HM219562–564, HM219568, JQ081320–341; G6pd: JQ081343–350. See Table S2 for more details).

For the nuclear genes, heterozygous states were identified as strong double peaks of similar height in both forward and reverse strands, or when the particular base corresponding to the dominant peak alternated on the two chromatograms [30].

### Microsatellite genotyping

The 225 samples used in this study were genotyped at twelve variable microsatellite loci using the multiplex sets and PCR conditions reported in [31]. Amplified DNA was analyzed for length variations on an ABI 3700 sequencer using GENEMAPPER 4.0 (Applied Biosystems).

### Mitochondrial and nuclear DNA analysis

#### Alignment, phasing and recombination

Sequences were aligned in BIOEDIT 7.0.9.0 [32] using the ClustalW algorithm. Haplotypes were identified using ARLEQUIN 3.11 [33]. For nuclear sequences, two alignments were created: first, heterozygous sites were coded using the IUPAC ambiguity codes (unphased dataset); and second, gametic phases of nuclear sequences were inferred using the Bayesian algorithm implemented in PHASE 2.1 [34] (phased dataset). Two runs were conducted for 1×10^4^ iterations with the default values.

Recombination in our nuclear genes was tested using the PHI test implemented in SPLITSTREE 4.11.3 [35] and the tree-based SBP and GARD methods [36] implemented online via the Datamonkey webserver [37].

#### Phylogenetic analysis

Phylogenetic reconstructions were performed using the maximum likelihood (ML) and Bayesian inference (BI) approaches. Analyses were first run independently on mitochondrial and nuclear loci and then on combined datasets (cytb/COI, bfibr/G6pd (both phased and unphased datasets) and cytb/COI/bfibr/G6pd). The most suitable model of DNA substitution for each locus and dataset was determined using MODELTEST 3.0 [38] according to the Akaike Information Criterion (AIC). PhyML 3.0 [39] was used to perform ML analyses with default parameters as starting values. Robustness of the tree was assessed by 1000 bootstrap replicates. Bayesian analyses were performed with MRBAYES 3.1.1. [40]. Metropolis-coupled Markov chain Monte Carlo (MCMC) sampling was performed with 5 chains run for 3×10^6^ generations with one tree sampled every 1000 generations. All trees obtained before the Markov chain reached stationary distribution (empirically determined by checking of likelihood values) were discarded as burn-in values. A 50% Majority-rule consensus tree was generated in PAUP 4.0b10 [41].

Median joining networks were performed with NETWORK 4.5.1.6 [42] to explore relationships among haplotypes for the mitochondrial (cytb/COI) and nuclear (bfibr/G6pd) datasets.

#### Genetic diversity and population differentiation

Haplotype (h) and nucleotide (π) diversities of the main lineages identified by phylogenetic analysis were estimated for the four loci independently using ARLEQUIN. Tables of nuclear allele frequency were computed with GENEPOP 4.0.11 [43] and frequency differences (genic differentiation) were tested for each nuclear locus and across all nuclear loci for all pairs of lineages containing more than three individuals with GENEPOP.

The net genetic distance between lineages was computed in MEGA 4.1 [44] under the Kimura two parameters (K2P model) for the cytb dataset. Genetic differentiation among lineages containing more than three individuals was quantified by computing pairwise differentiation Φ_ST_ for the mitochondrial dataset and *F*_ST_ for the nuclear dataset using ARLEQUIN.

A Mantel's test performed in ARLEQUIN on mitochondrial and nuclear datasets independently was used to assess the hypothesis of isolation by distance (IBD) among the four groups of populations by comparing pairwise geographic distance (log transformed) with pairwise (*F*_ST_/[1−*F*_ST_]).

#### Divergence time estimates

Divergence times of *L. neilli* and *L. edwardsi*, and of the main lineages of *L. neilli* (approximation of the time to the most recent common ancestor-TMRCA, [45]), were estimated using Bayesian inference, as implemented in the program BEAST 1.6.1 [46] on the cytb dataset. Three fossil-based calibration points were used: (i) the split between the tribe Phloemyini and the other tribes of Murinae at 12.3 Myr (we considered *Progonomys* [47] as the MRCA of extant murines and assigned the oldest record of *Progonomys* at 12.3 Myr to the split between the tribe Phloemyini and the other tribes of Murinae rather than the *Mus*/*Rattus* split [48], [49]) (ii) the divergence between *Apodemus mystacinus* and all the species of the subgenus *sylvaemus* (*A. flavicollis* and *A. sylvaticus*) at 7 Myr [50], [51]; and (iii) the *Otomyini/Arvicanthi* split at 5.7 Myr [52]. Sequences of *Batomys granti* (AY324458) (Phloemyini) and *Phloemys cumingi* (DQ191484) (Phloemyini), *Apodemus mystacinus* (AF159394), *Apodemus sylvaticus* (AB033695), *Apodemus flavicollis* (AB032853), *Otomys angoniensis* (AM408343) (Otomyini) and *Arvicanthis niloticus* (AF004569) (Arvicanthi) were added to our dataset to calibrate the tree and constrain the age of some nodes. Analyses were performed under the TN93+G substitution model (previously estimated by MODELTEST), a strict molecular clock and a constant size coalescent population model. These priors were selected because they better fit the data than any other molecular clock and population models according to the Bayes factors calculated to compare the models [53]. However, all tested models (strict or relaxed molecular clock combined to Bayesian Skyline coalescent population model, exponential growth coalescent population model or Yule process speciation model) produced similar divergence time estimates. Three independent runs with MCMC chain length of 1.5×10^8^ were performed, sampling every 1×10^4^ generations. Convergence of the chains to the stationary distribution was checked using TRACER 1.5 [54]. All BEAST computations were performed on the computational resource Bioportal at the University of Oslo (http://www.bioportal.uio.no).

### Microsatellites analysis

#### Genetic diversity

Genetic diversity was assessed by calculating expected (He) and observed (Ho) heterozygosities with ARLEQUIN and confirmation of Hardy-Weinberg equilibrium (HWE) was tested using GENEPOP for each locus separately and over all loci for each sampling site. The allelic richness (AR) was calculated using the rarefaction procedure implemented in FSTAT 2.9.3.2 [55]. Multi-locus *F*is was calculated for each population and adjusted for multiple tests using a Bonferroni's correction with FSTAT. The proportion of null alleles (NA) at each locus and for each population was estimated with FREENA [56] and genotypes were corrected using MICRO-CHECKER 2.2.3 [57]. Tests for linkage disequilibrium between loci for each sampling site were performed with GENEPOP.

#### Population structure

STRUCTURE 2.3.1 [58] was used to infer the number of populations (K) and assign individuals to genetic clusters independently of spatial sampling. Ten iterations were run for each value of K from 1 to 28 using an admixture model with a burn-in of 1×10^5^ and MCMC values of 1×10^6^. We used CLUMPP 1.1 [59] to average the results of multiple iterations for a given K. GENELAND 3.3.0 [60] was used to perform a spatial genetic analysis by integrating geographic and genetic information and to determine the most probable K. Ten runs of 1×10^5^ MCMC iterations were performed for K from 1 to 28 with a spatial coordinates uncertainty of 1 km. The posterior probability of population membership for each individual was determined using a burn-in of 3×10^4^ iterations. A visual output of the STRUCTURE and GENELAND results was generated using DISTRUCT [61].

Genetic differentiation among clusters containing more than three individuals was quantified by computing pairwise *F*_ST_ using ARLEQUIN.

IBD among the four groups of populations and within groups including at least four clusters (northeast and west) was tested by comparing pairwise geographic distance (log transformed) with pairwise (*F*_ST_/[1−*F*_ST_]) and (*R*_ST_/[1−*R*_ST_]) using ARLEQUIN and SPAGeDI 1.3 [62], respectively.

### Biogeographic analysis

#### Spatial Analysis of Vicariance

We used VIP (Vicariance Inference Program) [63] to localize the main isolating barriers within *L. neilli* distribution range. VIP uses a phylogenetic tree (ML tree of the combined dataset) and direct geographic information data to search for the disjunctions/barriers among sister groups.

#### Ancestral range reconstruction analyses

To infer ancestral areas of *L. neilli* populations and investigate the relative role of vicariance and dispersal in the evolutionary history of this species, we used a dispersal–extinction–cladogenesis (DEC) model of range evolution implemented in LAGRANGE [64]. The DEC model describes ancestor-descendant transitions between geographic ranges by processes of dispersal (range expansion), local extinction (range contraction), and cladogenesis (range subdivision/inheritance) and estimates likelihoods of ancestral states (range inheritance scenarios) [65]. This analysis was carried out on the chronogram inferred with BEAST limited to the ten lineages with four geographic areas (west, centre, north, northeast). We first performed an analysis without constraints to infer ancestral areas of each node of the tree. Then, to optimize the results obtained for the ancestral node of *L. neilli*, we constrained successively the area of this node to each combination of one, two, three or four areas (except for the combination centre+north because these two areas are not adjacent) and compared the global likelihood scores obtained for each area or combination.

#### ABC computations

**Figure 2:**
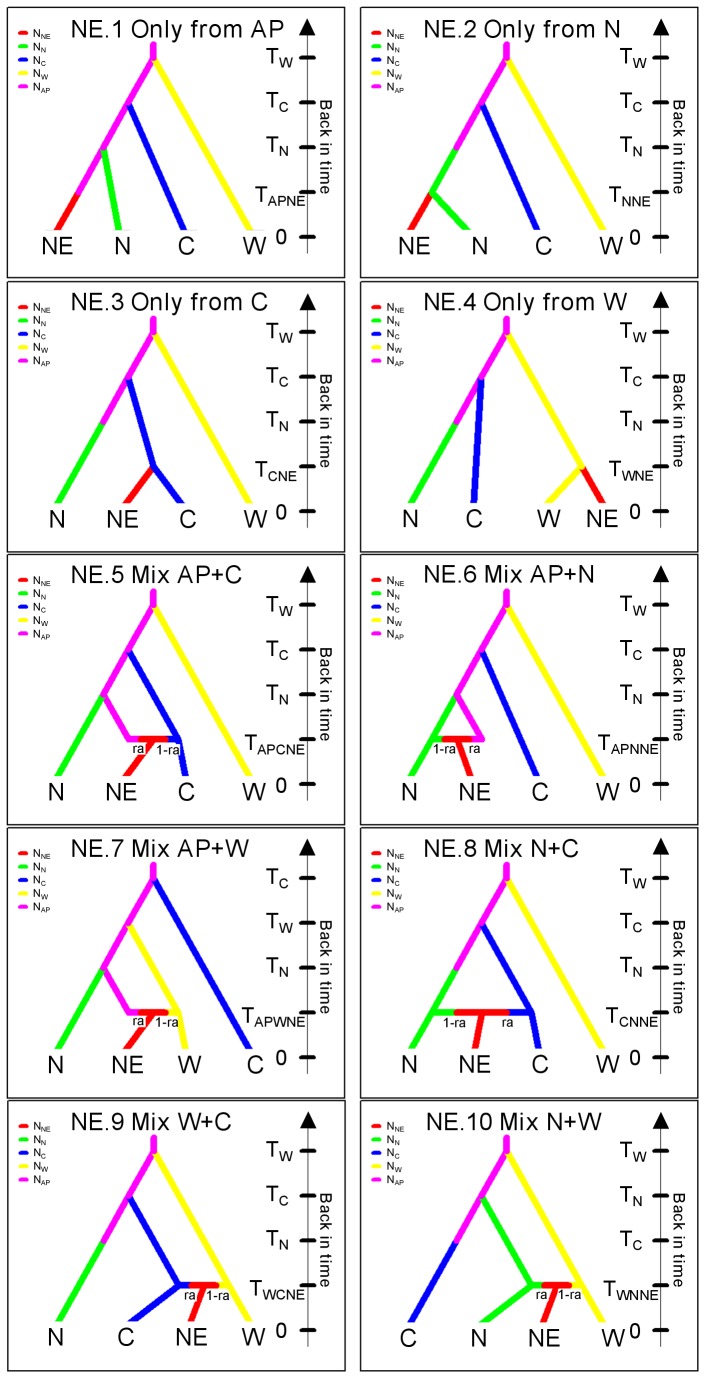
Schematic representations of ten competing scenarios designed to test the origin of the NE group by Approximate Bayesian Computation (ABC) analysis. N*i* corresponds to effective population sizes of each group. The time of events (T*i*), in numbers of generation, are not sorted by increasing times but are presented randomly as their prior distribution overlapped (Table 2). Several conditions were considered: T_NNE_<T_N_, T_CNE_<T_C_, T_WNE_<T_W_, T_APCNE_<T_C_, T_APNNE_<T_N_, T_APWNE_<T_W_, T_CNNE_<T_C_ and T_N_, T_WCNE_<T_W_ and T_C_, T_WNNE_<T_W_ and T_N_. Time 0 is the sampling date. Abbreviations are as follows: ancestral population (AP), western group (W), central group (C), northern group (N), northeastern group (NE), admixture rate (ra).

Approximate Bayesian computation implemented in the software DIYABC 1.0.4.45beta [66] was used to infer the evolutionary history of *L. neilli* combining our mitochondrial and microsatellite datasets. Several biogeographic scenarios were compared to test whether the current groups of populations originated from the fragmentation of a common ancestral population or from one of the current groups and whether admixture events occurred among populations. According to the phylogenetic trees and Bayesian clustering analysis, four groups of populations were used (west, centre, north, northeast) and the origin of each group was tested independently in a four-step analysis using 40 scenarios (Table 1). For each group, a set of ten scenarios was designed to test its origin (see Figure 2 for a schematic representation of these ten scenarios for the NE group, similar scenarios were used for the three other groups): either from a hypothetical ancestral population (AP) (scenario NE.1), from one of the current groups of populations (scenarios NE.2 to NE.4), or a mix of two populations (admixture, scenarios NE.5 to NE.10). Range and distribution of priors for parameters used to describe these scenarios (effective population size, time of splitting or merging events, and rates of admixture in the case of merging events) are found in Table 2. For the microsatellite dataset, all one-sample summary statistics available were used in addition to *F*_ST_ and classification index as two-sample statistics to compare observed and simulated datasets. For the mitochondrial dataset, we used all one-sample statistics and the number of segregating sites, the mean of pairwise differences (W) and *F*_ST_ as two-sample summary statistics.

**Table 1:** Scenarios used in the Approximate Bayesian computation (ABC) analysis.

Step	Scenario	Posterior probability	95% confidence interval
**Origin of WEST (W)**	**W.1 Only from AP**	**0.8526**	**[0.7655,0.9397]**
	W.2 Only from NE	0.0020	[0.0000,0.0047]
	W.3 Only from C	0.0005	[0.0000,0.0012]
	W.4 Only from N	0.0517	[0.0018,0.1016]
	W.5 Mix AP+C	0.0019	[0.0003,0.0036]
	W.6 Mix AP+NE	0.0044	[0.0008,0.0080]
	W.7 Mix AP+N	0.0823	[0.0282,0.1363]
	W.8 Mix C+NE	0.0001	[0.0000,0.0002]
	W.9 Mix C+N	0.0006	[0.0000,0.0014]
	W.10 Mix N+NE	0.0039	[0.0000,0.0081]
**Origin of CENTRE (C)**	**C.1 Only from AP**	**0.9824**	**[0.9553,1.0000]**
	C.2 Only from NE	0.0069	[0.0000,0.0207]
	C.3 Only from W	0.0045	[0.0000,0.0164]
	C.4 Only from N	0.0001	[0.0000,0.0003]
	C.5 Mix AP+W	0.0044	[0.0000,0.0123]
	C.6 Mix AP+NE	0.0007	[0.0000,0.0019]
	C.7 Mix AP+N	0.0009	[0.0000,0.0025]
	C.8 Mix W+NE	0.0000	[0.0000,0.0000]
	C.9 Mix W+N	0.0000	[0.0000,0.0001]
	C.10 Mix N+NE	0.0000	[0.0000,0.0000]
**Origin of NORTH (N)**	**N.1 Only from AP**	**0.7542**	**[0.4905,1.0000]**
	N.2 Only from NE	0.0426	[0.0000,0.1170]
	N.3 Only from C	0.0001	[0.0000,0.0002]
	N.4 Only from W	0.0000	[0.0000,0.0000]
	N.5 Mix AP+C	0.0285	[0.0000,0.0722]
	N.6 Mix AP+NE	0.0179	[0.0000,0.0456]
	N.7 Mix AP+W	0.1070	[0.0000,0.2716]
	N.8 Mix C+NE	0.0130	[0.0000,0.0339]
	N.9 Mix C+W	0.0028	[0.0000,0.0078]
	N.10 Mix NE+W	0.0338	[0.0000,0.0920]
**Origin of NORTHEAST (NE)**	**NE.1 Only from AP**	**0.5630**	**[0.4342,0.6918]**
	NE.2 Only from N	0.0046	[0.0008,0.0084]
	NE.3 Only from C	0.0042	[0.0000,0.0085]
	NE.4 Only from W	0.0051	[0.0000,0.0103]
	NE.5 Mix AP+C	0.2130	[0.1063,0.3197]
	NE.6 Mix AP+N	0.0283	[0.0131,0.0435]
	NE.7 Mix AP+W	0.1450	[0.0716,0.2184]
	NE.8 Mix C+N	0.0085	[0.0021,0.0148]
	NE.9 Mix C+W	0.0242	[0.0051,0.0433]
	NE.10 Mix N+W	0.0041	[0.0012,0.0070]

Schematic representations of these scenarios are given in Figure 2. The best scenario for each step is emboldened.

**Table 2:** Prior distribution of parameters used in our ABC analysis.

Parameter	Distribution	Min.	Max.	Step
**Effective population size**				
N_AP_, N_W_, N_C_, N_N_, N_NE_	uniform	10	50000	1
**Time of events (in generations backward in time)**				
T_i_ [Conditions: T_NNE_<T_N_, T_CNE_<T_C_, T_WNE_<T_W_, T_APCNE_<T_C_, T_APNNE_<T_N_, T_APWNE_<T_W_, T_CNNE_<T_C_ and T_N_, T_WCNE_<T_W_ and T_C_, T_WNNE_<T_W_ and T_N_]	log-uniform	10	5000000	1
**Admixture rate (ra)**	uniform	0.001	0.999	0.001
**Microsatellites: Mutation model parameters**				
Mean mutation rate	uniform	0.0001	0.001	
Mean coefficient p	uniform	0.1	0.3	
Mean SNI rate	log-uniform	0	0	
**Mitochondrial DNA: Mutation model parameters**				
Mean mutation rate	uniform	1×10^−8^	1×10^−7^	
Mean coefficient kappa	uniform	0.050	20.00	

For each scenario, 5×10^5^ datasets were simulated to build a reference table. To check if the combination of scenarios and prior distributions of their parameters were able to generate datasets similar to the observed one, a principal component analysis (PCA) was performed on the first 10000 simulated datasets of the reference table in the space of summary statistics. To determine the most likely scenario, the normalized Euclidean distances between each simulated dataset of the reference table and the observed dataset was then computed and 1% of the closest simulated datasets were used to estimate the relative posterior probability (with 95% confidence intervals) of each scenario with a logistic regression [67]. For each set of scenarios, the most likely scenario was the one with the highest posterior probability value and non-overlapping 95% confidence intervals. Finally, the posterior density distributions of the effective population size of each group were estimated from 1% of the closest datasets simulated according to the most likely scenario obtained for each step.

## Results

### Mitochondrial and nuclear DNA analysis

#### Sequence variation

Fragments of 900 and 713 bp were obtained for cytb and COI genes, respectively. No stop codons, indels or heterozygous sequences were observed in these mitochondrial coding genes, supporting the mitochondrial origin of the sequences amplified. A total of 37 cytb haplotypes and 25 COI haplotypes were identified among our datasets. The cytb gene contained 158 (231 in the dataset including outgroup sequences) variable sites, whereas the COI gene contained 85 (138) variables sites.

For the bfibr gene, fragments of 740 bp were obtained and no indels were observed in the alignment. The G6pd sequences obtained varied in length from 678 to 690 bp and the final alignment included three indels of 1, 2 and 10 bp. A total of 31 bfibr alleles and eight G6pd alleles were identified among our datasets. The bfibr gene contained 27 (49) variables sites, whereas the G6pd gene contained 17 (29) variables sites.

Recombination was detected via a PHI test (p = 0.004) and GARD/SBP tests at position 386 for the bfibr gene. G6pd sequences did not present evidence of recombination, according to the PHI test (p = 1.0) and GARD/SBP tests. Therefore, we performed all phylogenetic trees including the bfibr gene with alignment containing only positions 1–385 of the bfibr gene.

#### Phylogenetic analyses

**Figure 3:**
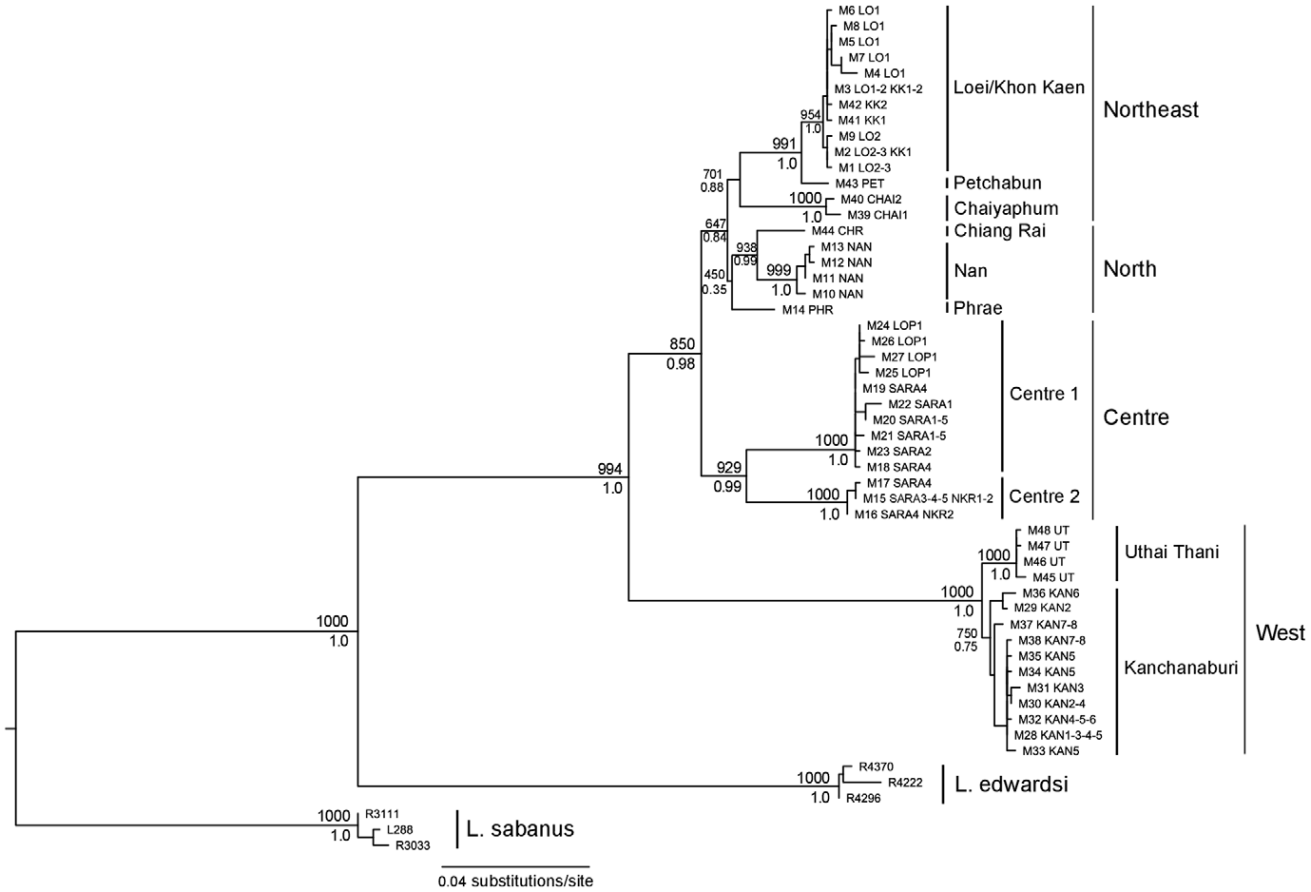
Maximum likelihood tree based on mitochondrial dataset (GTR+G). Bootstrap support (1000 replicates) and posterior probabilities of nodes are indicated above and below the branches, respectively. Node support values from within lineages were removed for clarity.

Mitochondrial loci were first analyzed separately; they yielded low-supported but congruent phylogenies (data not shown). They were then combined in a single matrix. The mitochondrial phylogenetic tree (Figure 3) clearly indicated that the sister species of *L. neilli* is represented by *L. edwardsi* haplotypes and that all analyzed individuals belong to the *L. neilli* monophyletic group. The ML and Bayesian analysis gave congruent results. The haplotypes of *L. neilli* clustered into ten well-supported and geographically well-structured lineages: Loei/Khon Kaen (localities LO1-2-3, KK1-2), Petchabun (PET), Chaiyaphum (CHAI1-2), Chiang Rai (CHR), Nan (NAN), Phrae (PHR), Centre1 (SARA1-2-4-5, LOP1), Centre2 (SARA3-4-5, NKR1-2), Uthai Thani (UT), and Kanchanaburi (KAN1-2-3-4-5-6-7-8). These results are congruent with those obtained in Latinne *et al.* [25] and four new lineages are identified: Petchabun, Chaiyaphum, Chiang Rai and Uthai Thani. All these lineages are allopatric with the exception of the Centre1 and Centre2 lineages which both contain haplotypes from the same localities (SARA4-5). The western lineages (Uthai Thani and Kanchanaburi) form a well-supported group (BS = 100%-BP = 1.0), separated from all the other regions nested in a single well-supported group (BS = 85%-BP = 0.98). Within this group, three sub-groups are observed: a northeastern sub-group (BS = 70%-BP = 0.86) (Loei/Khon Kaen, Petchabun and Chaiyaphum) sister to a weakly supported northern sub-group (BS = 45%-BP = 0.35) (Chiang Rai, Nan and Phrae) and finally a central sub-group (BS = 93%-BP = 0.99) including the Centre1 and Centre2 lineages.

**Figure 4:**
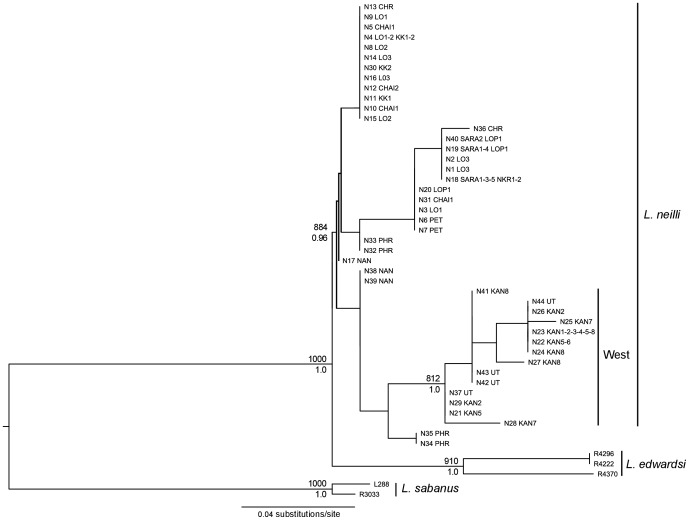
Maximum likelihood tree based on nuclear unphased dataset (GTR+G). Bootstrap support (1000 replicates) and posterior probabilities of nodes are indicated above and below the branches, respectively. Only nodes with BS>750 and/or BP>0.95 were indicated.

Due to the low variability of our nuclear genes they were combined in a single matrix. For both phased and unphased datasets, the nuclear phylogenetic tree (Figure 4) supported the monophyly of the three *Leopoldamys* species; however, intraspecific relationships within *L. neilli* were less clear than for mitochondrial dataset. Only one lineage is well-supported (BS = 81%–BP = 1.0) and includes all samples from western Thailand (UT, KAN1-2-3-4-5-6-7-8).

**Figure 5:**
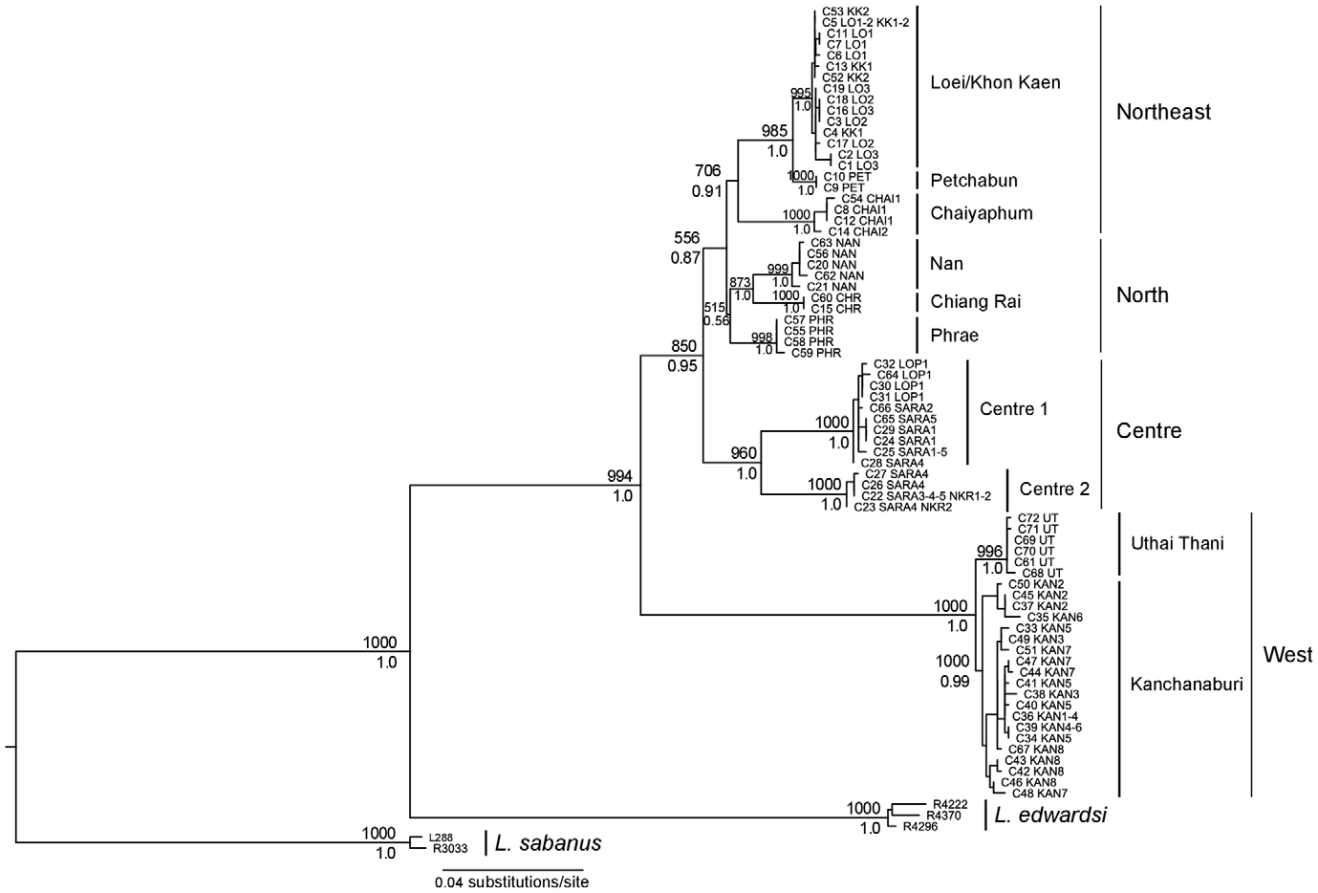
Maximum likelihood tree based on combined dataset (GTR+G). Bootstrap support (1000 replicates) and posterior probabilities of nodes are indicated above and below the branches, respectively. Node support values from within lineages were removed for clarity.

The ML and Bayesian trees combining nuclear and mitochondrial datasets (Figure 5) recovered exactly the same topology as the mitochondrial tree with better support for some nodes.

**Figure 6:**
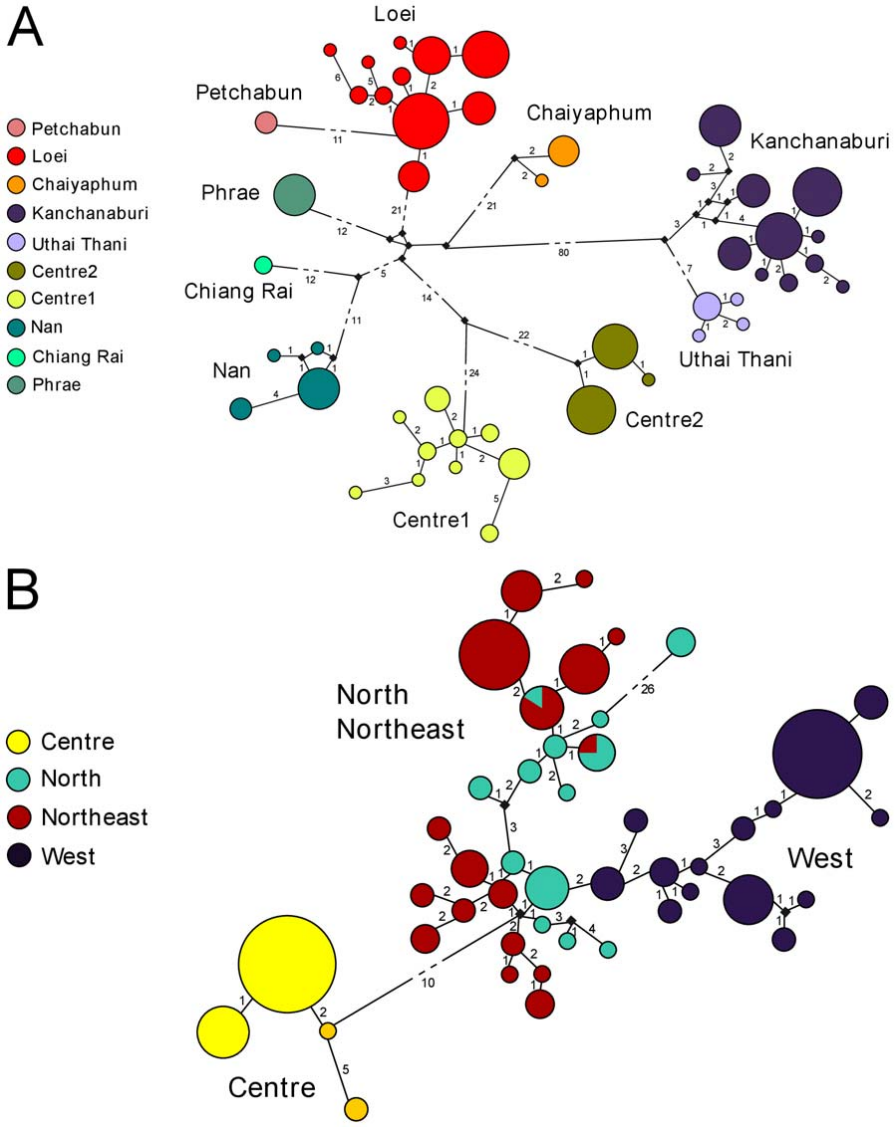
Median-joining networks based on mitochondrial (A) and nuclear (B) datasets. Circles represent haplotypes obtained in this study and the circle size is proportional to the number of individuals sharing a haplotype. Squares represent median vectors. Numbers on branches represent the number of mutational steps between haplotypes.

The median joining network of mitochondrial haplotypes confirmed the results obtained in the phylogenetic trees and retrieved the same ten haplogroups (Figure 6A). They are isolated by a high number of mutational steps, in particular the Uthai Thani and Kanchanaburi haplogroups (more than 80 steps). The network of nuclear alleles is less clear but the western (UT, KAN1-2-3-4-5-6-7-8) and central (SARA1-2-3-4-5, NKR1-2, LOP1) alleles are distinctly separated from the northern (NAN, PHR, CHR) and northeastern (LOEI1-2-3, KK1-2, PET, CHAI1-2) alleles (Figure 6B).

#### Genetic diversity and population differentiation

Haplotype diversities within lineages are quite low (Table 3), ranging from 0.0 and 0.775 for mitochondrial markers and from 0.0 to 1.0 for nuclear markers. Nucleotide diversities (expressed as percentages) varied from 0.0 to 0.33 and from 0.0 to 0.73 for mitochondrial and nuclear markers respectively (Table 3).

**Table 3:** Diversity estimates of the main lineages of *L. neilli* identified by phylogenetic analyses.

Marker	Lineage	Sample size	Number of haplotypes	Haplotype diversity h ± SD	Nucleotide diversity (percentage) π ± SD
**cytb**	Overall	225	37	0.946±0.005	4.505±2.174
	Loei/KK	65	6	0.727±0.039	0.206±0.134
	Petchabun	3	1	0.000±0.000	0.000±0.000
	Chaiyaphum	7	2	0.286±0.196	0.102±0.093
	Chiang Rai	2	1	0.000±0.000	0.000±0.000
	Nan	16	4	0.517±0.132	0.174±0.125
	Phrae	11	1	0.000±0.000	0.000±0.000
	Centre 1	22	7	0.762±0.077	0.214±0.149
	Centre 2	29	3	0.549±0.041	0.119±0.090
	Uthai Thani	8	4	0.643±0.184	0.132±0.112
	Kanchanaburi	62	8	0.775±0.028	0.332±0.195
**COI**	Overall	225	25	0.885±0.011	3.032±1.485
	Loei/KK	65	6	0.351±0.073	0.082±0.074
	Petchabun	3	1	0.000±0.000	0.000±0.000
	Chaiyaphum	7	2	0.286±0.196	0.042±0.057
	Chiang Rai	2	1	0.000±0.000	0.000±0.000
	Nan	16	2	0.325±0.125	0.046±0.054
	Phrae	11	1	0.000±0.000	0.000±0.000
	Centre 1	22	4	0.636±0.067	0.186±0.134
	Centre 2	29	1	0.000±0.000	0.000±0.000
	Uthai Thani	8	1	0.000±0.000	0.000±0.000
	Kanchanaburi	62	6	0.534±0.068	0.177±0.125
**bfibr**	Overall	104	31	0.906±0.010	0.747±0.399
	Loei/KK	22	8	0.716±0.062	0.547±0.309
	Petchabun	3	2	0.600±0.129	0.730±0.473
	Chaiyaphum	7	3	0.648±0.081	0.508±0.306
	Chiang Rai	2	4	1.000±0.177	0.692±0.510
	Nan	6	6	0.848±0.074	0.384±0.246
	Phrae	6	4	0.651±0.133	0.391±0.248
	Centre 1	12	4	0.612±0.071	0.232±0.156
	Centre 2	12	2	0.159±0.094	0.021±0.034
	Uthai Thani	6	3	0.621±0.118	0.510±0.311
	Kanchanaburi	28	10	0.634±0.069	0.456±0.263
**G6pd**	Overall	104	8	0.819±0.011	0.837±0.445
	Loei/KK	22	3	0.317±0.082	0.101±0.086
	Petchabun	3	2	0.533±0.172	0.077±0.085
	Chaiyaphum	7	1	0.000±0.000	0.000±0.000
	Chiang Rai	2	2	0.500±0.265	0.073±0.090
	Nan	6	1	0.000±0.000	0.000±0.000
	Phrae	6	1	0.000±0.000	0.000±0.000
	Centre 1	12	1	0.000±0.000	0.000±0.000
	Centre 2	12	1	0.000±0.000	0.000±0.000
	Uthai Thani	6	1	0.000±0.000	0.000±0.000
	Kanchanaburi	28	2	0.321±0.065	0.047±0.053

The table of nuclear allele frequency showed a high genetic heterogeneity among lineages (Table S4). Exact tests of genic differentiation computed across all pairs of lineages containing more than three individuals were highly significant at each locus (except for the pair Loei/Khon Kaen-Chaiyaphum (p = 0.10) for the bfibr gene and for the pair Uthai Thani-Kanchanaburi (p = 0.12) for the G6pd gene), as well as over all nuclear loci. Moreover, the Phrae and Nan lineages shared a single G6pd allele and the Centre1 and Centre2 lineages also.

The analysis of pairwise Φ_ST_ and *F*_ST_ values confirmed a high population differentiation (Table 4) and most comparisons were statistically significant. Nonsignificant genetic differentiation was only observed for nuclear datasets between geographically close lineages: Loei/Khon Kaen and Chaiyaphum lineages (*F*_ST_ = 0.024, p = 0.18), Centre1 and Centre2 lineages (*F*_ST_ = 0.053, p = 0.12). Pairwise Φ_ST_ and *F*_ST_ values were particularly high between western (Kanchanaburi, Uthai Thani) and central (Centre1, Centre2) lineages (from 0.96 to 0.99 for Φ_ST_ and from 0.66 to 0.84 for *F*_ST_). These results are confirmed by the high genetic divergence (net K2P distance, cytb dataset) observed among lineages (Table S5). Three levels of genetic distance are observed among our dataset: around 7.8% of K2P distance (7.4–8.8%) between western lineages and all the other lineages, around 3.4% (2.8–4.3%) between central and northern/northeastern lineages and finally around 1.5% (0.7–2.3%) between northern and northeastern lineages.

**Table 4:** Pairwise *F*_ST_ among lineages containing more than three individuals calculated on mitochondrial (below diagonal) and nuclear (above diagonal) datasets.

	Loei/KK	Chaiyaphum	Nan	Phrae	Centre1	Centre2	Uthai Thani	Kanchanaburi
**Loei/KK**		0.02415*	0.32828	0.38524	0.43158	0.50245	0.57973	0.59657
**Chaiyaphum**	0.95316		0.28283	0.33639	0.45774	0.62184	0.56425	0.60009
**Nan**	0.95209	0.96022		0.33319	0.51424	0.57714	0.46070	0.53737
**Phrae**	0.94944	0.98703	0.96337		0.50752	0.69940	0.48711	0.58286
**Centre1**	0.96136	0.95441	0.95402	0.96004		0.05334*	0.71862	0.66498
**Centre2**	0.97369	0.98816	0.98044	0.99064	0.96479		0.84636	0.72141
**Uthai Thani**	0.98120	0.98961	0.98545	0.99598	0.97744	0.99362		0.42344
**Kanchanaburi**	0.96981	0.96346	0.96474	0.96595	0.96525	0.97190	0.76411	

Non significant values are marked with asterisks (significance level = 0.05).

The Mantel's test was nonsignificant for both mitochondrial (*r* ^2^ = 0.09; p = 0.66) and nuclear datasets (*r* ^2^ = 0.56; p = 0.17). This result is consistent with no relationship between geographic distance and genetic distance.

#### Divergence time estimates

**Figure 7:**
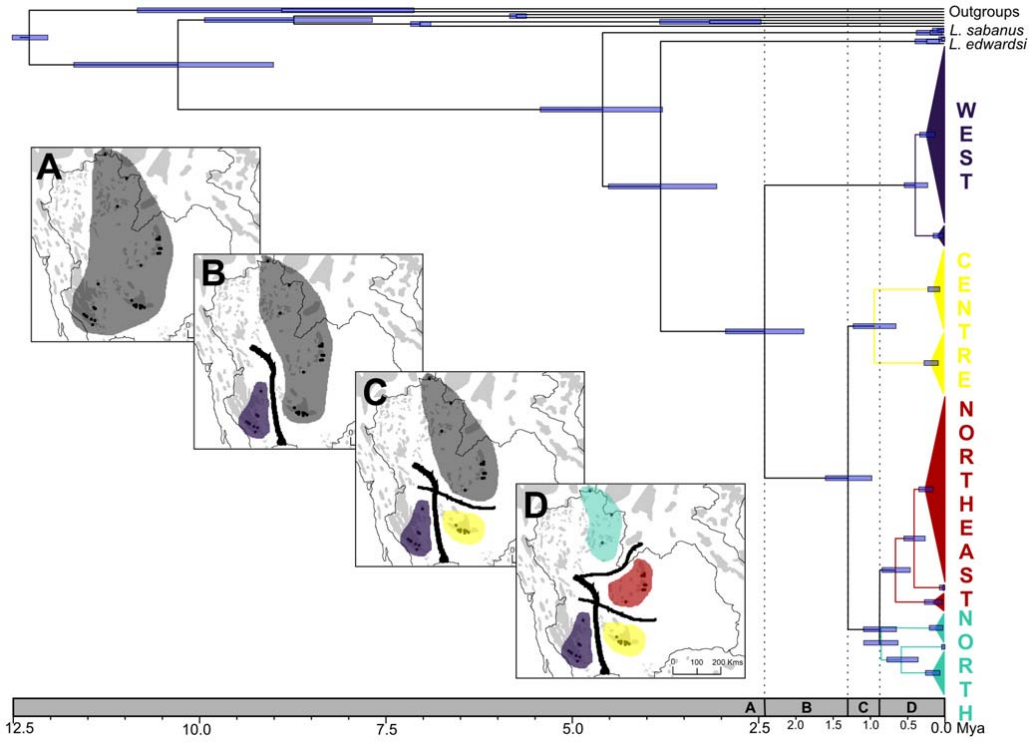
Dating of the most recent common ancestors with 95% HPD (blue node bars) computed with BEAST and graphical representation of the biogeographic scenario of *L. neilli* according to four time periods (A, B, C, D) inferred by VIP, LAGRANGE and DIYABC. The four maps depict the hypothetical distribution of *L. neilli* ancestral population (dark grey) (obtained with LAGRANGE), western (mauve), central (yellow), northern (light blue) and northeastern (red) groups and the localisation of barriers (black lines) leading to three vicariant events (obtained with VIP).

Divergence time estimates are given in Table 5 and Figure 7. The TMRCA of *L. neilli* and *L. edwardsi* was estimated to occur at around 3.8 Myr. The TMRCA of *L. neilli* was dated around 2.4 Myr. The TMRCA of (centre+north+northeast) was estimated around 1.3 Myr while the TMRCA of (north+northeast) was dated around 0.9 Myr.

**Table 5:** TMRCA (mean values and 95% HPD) of the clades observed in phylogenetic trees.

	Mean (kyr)	95% HPD (kyr)
TMRCA (*L. neilli*+*L. edwardsi*)	3785	3053–4499
TMRCA (*L. neilli*)	2395	1892–2935
TMRCA (Centre+North+Northeast)	1282	980–1598
TMRCA (North+Northeast)	860	653–1094
TMRCA (Northeast)	647	468–845
TMRCA (North)	837	602–1068
TMRCA (Centre)	932	655–1226
TMRCA (West)	381	229–541
TMRCA (Loei/KK)	251	159–352
TMRCA (Petchabun)	28	0–74
TMRCA (Chaiyaphum)	150	35–274
TMRCA (Chiang Rai)	14	0–45
TMRCA (Nan)	157	67–261
TMRCA (Phrae)	113	31–211
TMRCA (Centre1)	178	92–278
TMRCA (Centre2)	142	70–228
TMRCA (Uthai Thani)	83	21–157
TMRCA (Kanchanaburi)	233	134–336

### Microsatellites analysis

#### Genetic diversity

Analysis of microsatellite genetic diversity showed that observed heterozygosity ranged from 0.43 to 0.93, and allelic richness (based on one diploid individual) ranged from 1.41 to 1.79 (Table 6). Tests for HWE showed deviation from the expected frequencies for three populations: KAN2, KAN4 and KAN5. In the KAN2 and KAN5 populations, the homozygote excess could be due to NA at single loci (32% at locus mLn05 in Kan2 and 29% at locus mLn06 in Kan5) but NA were not detected in KAN4 population. None of the inbreeding coefficient (*F*is) was significant at population level. Only one pair of loci (mLn04 and mLn12) showed significant linkage disequilibrium in two populations (NAN, KK2) after Bonferroni's correction.

**Table 6:** Genetic diversity within *L. neilli* populations based on microsatellite dataset.

Province	Locality ID	n	Ho	He	HWE	AR	*F*is
**Loei**	LO1	17	0.80±0.17	0.79±0.10	0.87	1.79	−0.018
	LO2	14	0.74±0.16	0.75±0.12	0.23	1.75	0.008
	LO3	9	0.75±0.17	0.70±0.09	0.82	1.70	−0.068
**Khon Kaen**	KK1	10	0.68±0.15	0.73±0.08	0.058	1.73	0.068
	KK2	15	0.66±0.22	0.63±0.14	0.32	1.63	−0.048
**Chaiyaphum**	CHAI1	6	0.74±0.24	0.61±0.18	1.00	1.61	−0.241
	CHAI2	1	1.00±0.00	1.00±0.00	−	1.67	−
**Petchabun**	PET	3	0.70±0.28	0.68±0.17	0.99	1.63	−0.022
**Nan**	NAN	16	0.71±0.17	0.71±0.09	0.27	1.71	0.010
**Phrae**	PHR	11	0.48±0.26	0.51±0.24	0.18	1.51	0.065
**Chiang Rai**	CHR	2	0.85±0.24	0.70±0.15	1.00	1.58	−0.360
**Saraburi**	SARA1	8	0.53±0.18	0.63±0.15	0.11	1.58	0.167
	SARA2	2	0.93±0.19	0.71±0.13	1.0	1.42	−0.529
	SARA3	1	1.00±0.00	1.00±0.00	-	1.58	-
	SARA4	15	0.70±0.23	0.71±0.17	0.39	1.71	0.022
	SARA5	5	0.60±0.31	0.63±0.21	0.98	1.63	0.050
**Nakhon Ratchasima**	NKR1	1	1.00±0.00	1.00±0.00	-	1.67	-
	NKR2	14	0.66±0.22	0.64±0.15	0.54	1.63	−0.041
**Lopburi**	LOP1	5	0.65±0.26	0.62±0.19	0.99	1.62	−0.061
**Uthai Thani**	UT	8	0.60±0.23	0.57±0.08	0.76	1.57	−0.057
**Kanchanaburi**	KAN1	1	1.00±0.00	1.00±0.00	-	1.75	-
	KAN2	12	0.63±0.27	0.61±0.15	**<0.001**	1.61	−0.031
	KAN3	2	0.82±0.34	0.79±0.20	1.0	1.72	−0.059
	KAN4	15	0.69±0.12	0.73±0.08	**0.047**	1.73	0.054
	KAN5	7	0.71±0.17	0.77±0.11	**0.037**	1.77	0.085
	KAN6	3	0.63±0.26	0.61±0.14	1.00	1.46	−0.030
	KAN7	16	0.43±0.31	0.44±0.25	0.40	1.41	0.037
	KAN8	6	0.58±0.16	0.58±0.11	1.00	1.48	−0.012

#### Population structure

**Figure 8:**
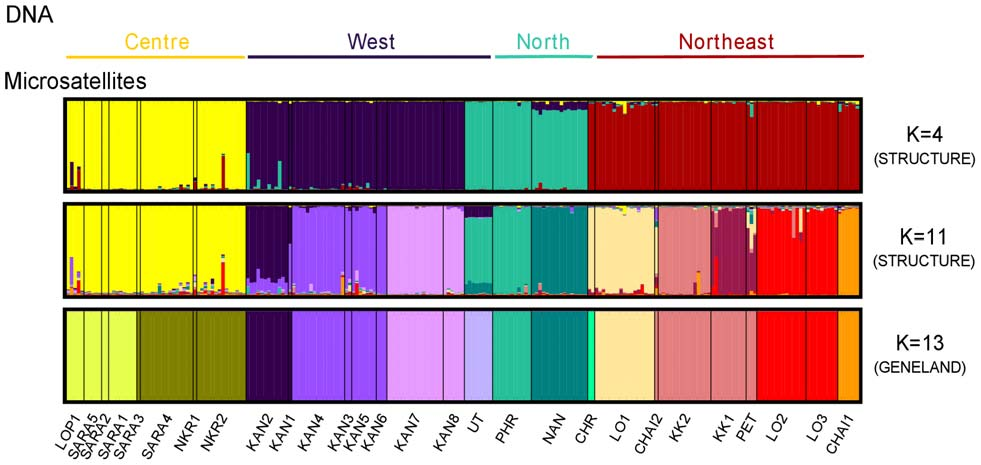
Population structure estimated using STRUCTURE (K = 4, K = 11) and GENELAND (K = 13). Each individual is represented by a vertical line partitioned into K colour segments, the length of each colour being proportional to the estimated membership coefficient.

The STRUCTURE output was interpreted using the *ΔK* method described by Evanno *et al.* [68]. The highest *ΔK* was found at K = 11 with a second peak at K = 4 (Figure S2). At K = 4, the four clusters correspond broadly to the four sub-groups observed in phylogenetic trees (west, centre, north, northeast) with the exception of UT and CHR samples included in the north and northeast clusters respectively (Figure 8). At K = 11, three of these clusters (west, north, northeast) are subdivided into ten clusters and inconsistencies with phylogenetic trees were still observed for UT and CHR samples which clustered with PHR and LO1+CHAI2 samples respectively (Figure 8). Few individuals were identified as possessing apparent admixed genotypes between central and northeastern clusters (in LOP1 and NKR2 populations) and between western and northern clusters (in KAN2 population).

Nine out of ten GENELAND runs gave a K of 13 clusters; while the other one gave a K of 12 clusters. As the mean posterior densities of the K = 13 runs were the highest, we selected K = 13 as the most probable number of clusters. These 13 “geneland clusters” were slightly different from the 11 “structure clusters”. Most of them were congruent with the ten lineages identified in the phylogenetic trees and revealed a finer population structure within Kanchanaburi and Loei/Khon Kaen lineages (Figure 8).

All pairwise *F*_ST_ values among “geneland clusters” were significantly higher than zero, indicating a strong genetic differentiation (Table S6).

IBD was not detected among the four groups of populations using both *F*_ST_ and *R*_ST_ (*r* ^2^ = 0.13; p = 0.33 and *r* ^2^ = 0.09; p = 0.34, respectively) and within the west (*r* ^2^ = 0.13; p = 0.37 and *r* ^2^ = 0.38; p = 0.25, respectively) and northeast (*r* ^2^ = 2.5×10^−4^; p = 0.33 and *r* ^2^ = 0.03; p = 0.34, respectively) groups.

### Biogeographic analysis

#### Spatial Analysis of Vicariance

VIP identified three main barriers within the studied area (Figure 7). The oldest barrier, isolating the western populations from the others, coincides exactly with the Central Plain of Thailand.

#### Ancestral range reconstruction analyses

LAGRANGE estimated the likelihoods of all possible ancestral range reconstructions for each node of the *L. neilli* tree. The most likely area of *L. neilli* ancestral population encompasses the four regions (west+centre+north+northeast) (relative probability = 1) (Figure 7). The likelihood scores of other areas or combinations of areas for the ancestral range of *L. neilli* are significantly different (more than 3-log likelihood units between the most likely area and the remaining likelihood scores). Several vicariant events progressively fragmented this large population and isolated the four regions. The ancestral range of the node containing the central, northern and northeastern lineages includes the three regions (centre+north+northeast) (relative probability = 1). Finally, the ancestral range of the node containing the northern and northeastern lineages encompasses these two regions (north+northeast) (relative probability = 1).

#### ABC computations

The PCA performed on the first 10000 simulated datasets of the reference table in the space of summary statistics revealed that the observed dataset is clearly surrounded by simulated datasets, indicating that our model was able to produce datasets similar to the observed one (data not shown).

The logistic regressions performed on 1% of the closest simulated datasets allow distinguishing unambiguously the most likely of all tested scenarios at each step and revealed the origin of the four groups of populations. The most likely scenarios (highest posterior probability) of each step clearly indicated that all of the four groups originate from a common ancestral population and not from one of the current populations and that admixture events did not occur (Table 1 and Figure S3). These results corroborated the hypothesis of the fragmentation of a widespread ancestral population (vicariant hypothesis). The posterior parameter estimation indicated that the effective population size of this ancestral population was several times higher than those of the current groups of populations. Moreover we consistently observed that the western group has the largest effective population size, followed by the northeastern group, the central group and finally the northern group has the smallest effective population size (N_W_>N_NE_>N_C_>N_N_) (Table S7).

## Discussion

### Mitochondrial vs. nuclear phylogeographic structures of *L. neilli*

Three kind of molecular markers with various evolutionary rates and different modes of inheritance were used in this study and depicted an exhaustive picture of the phylogeographic structure of *L. neilli*.

Due to their slow evolutionary rate and their higher coalescent time, the use of nuclear DNA sequences in phylogeographic studies has been limited. However the present study confirms the interest to combine several nuclear introns to gain more resolution. Indeed the intron 7 of the bfibr gene used alone in Latinne *et al.* [25] did not show any phylogeographic signal but the phylogenetic tree combining this intron with the intron 1 of the G6pd gene (Figure 4) clearly confirmed the separation of the western populations from all the others. Furthermore, the nuclear haplotype network (Figure 6B) supported the high divergence among western, central and northern/northeastern populations. These results indicate the ability of nuclear introns to detect old vicariant events at the intraspecific level for *L. neilli*. The exact tests of genic differentiation among lineages also confirmed a high genetic heterogeneity of nuclear allele frequency at a finer scale.

The mtDNA markers corroborated the results obtained with nuclear introns and supported a large-scale population structure of four main groups (west, centre, north, northeast). Moreover they also detected more recent isolations among populations and indicated a strong finer structure within each of these groups: ten well-supported and geographically well-structured lineages of *L. neilli* are observed within Thailand (Figures 3, 6A).

Finally the microsatellite markers also detected the two levels of population structure using the *ΔK* method (Figures 8, S2). Microsatellites and mtDNA gave broadly congruent results and due to their high levels of polymorphism, microsatellites revealed a micro-fragmentation of the genetic diversity within western and northeastern regions that was not detected by mtDNA. Only two minor discrepancies were observed between these markers. The microsatellite-based clustering of samples from Uthai Thani (UT) with those from Phrae (PHR) (northern group) in the STRUCTURE analysis conflicts with the mitochondrial and nuclear phylogenetic analyses indicating a strong link between samples from Uthai Thani and Kanchanaburi (western group). The fact that mitochondrial and nuclear genes sequences gave a similar result points towards an accuracy problem of microsatellite markers (e.g. size homoplasy) rather than a real cytonuclear conflict. Moreover, the GENELAND clustering analysis that integrates the spatial distribution of samples did not recover the UT+PHR cluster (Figure 8). An additional discordance was observed for samples from Chiang Rai (CHR), which clustered with northeastern samples (LO1+CHAI2) in STRUCTURE analysis (Figure 8), but were nested within the northern group in phylogenetic trees (Figures 3, 5). The table of nuclear allele frequency (Table S4) also indicates a link between CHR and northeastern lineages. However, due to the low sample size for this locality, it is not possible to draw a certain conclusion on this possible cytonuclear conflict.

The admixed genotypes between central and northeastern clusters identified in LOP1 and NKR2 populations and between western and northern clusters in KAN2 (Figure 8) most probably reflect the co-ancestry shared by samples of these clusters. Few bfibr alleles are also shared among some of the four groups of populations (Table S4) and they also probably represent polymorphism maintained from the common ancestral population.

In conclusion the present study confirms the benefit of using several independent and complementary genetic markers to obtain a comprehensive and reliable picture of *L. neilli* evolutionary history at several levels of resolution.

The congruent patterns of mitochondrial and nuclear markers indicated a strong population fragmentation for *L. neilli*. The absence of geographical overlap in the mtDNA haplotype distribution among lineages (except for Centre1 and Centre2 lineages) coupled with the high *F*_ST_ values and genetic distances among them revealed a high genetic divergence and low gene flows among lineages. This high degree of genetic differentiation is confirmed by the old divergence times estimated among lineages as the TMRCA of *L. neilli* was approximated around 2.4 Myr. Such deep genealogical divergence among geographically close lineages is quite surprising and denotes a high population fragmentation related to the patchy distribution of limestone karsts and a close association of *L. neilli* with this habitat. Similar phylogeographic patterns are rare among rodents but have been observed for a few other rodent species strongly dependent on rocky and karstic habitats in various regions of the world, such as the Laotian rock rat *Laonastes aenigmamus* in Central Lao PDR [69], the Martino's vole *Dinaromys bogdanovi* in the western Balkans [70], [71] and the Allegheny woodrat *Neotoma magister* in northeastern America [72]. Our study suggests that the spatial isolation of karsts prevents extensive migration among lineages of *L. neilli*. These results confirm the endemicity of *L. neilli* to limestone karsts previously described in the literature [17], [19], [25].

### Evolutionary history of *L. neilli*

The divergence between *L. neilli* and *L. edwardsi* was estimated to occur during Pliocene, around 3.8 Myr. Several splits subsequently occurred within *L. neilli* population throughout Quaternary. Due to the potential inaccuracy of temporal estimates based on molecular data across different evolutionary scales [73], [74], these estimates should be considered as a general approximation rather than as exact dating. These dates are consistent with fossil evidence attesting the widespread presence of the genus *Leopoldamys* in Indochina during Plio-Pleistocene. The oldest occurrence of *Leopoldamys* (*L. minutus* n. sp., an extinct species) has been reported in cave deposits from Late Pliocene to Early Pleistocene in the Kanchanaburi and Saraburi provinces, Thailand [75]. Fossils of *L. edwardsioides*, a predecessor of *L. edwardsi*, from Early Pleistocene have also been identified in southern China [76].

The evolutionary context of the divergence between *L. neilli* and *L. edwardsi* is a matter of debate. Two main alternative models of speciation of cave animals have been proposed in the literature: the Climatic Relict Hypothesis (CRH) [7], an allopatric speciation model; and the Adaptive Shift Hypothesis (ASH) [8], [9], a divergence with gene flow speciation model (see Introduction for more details). Even if these two modes of speciation have been proposed for troglobionts, i.e., permanent residents of subterranean habitat without link with the surface [21], it could be interesting to attempt to use it to explain the speciation of a mammal subtroglophilic species.

The old divergence between *L. neilli* and *L. edwardsi* that occurred a long time before Pleistocene environmental instability and the present parapatric/partially sympatric distribution of these two sister species in northern and northeastern Thailand [77] could support the ASH. According to this model, populations of a forest *Leopoldamys* ancestor could have invaded cave ecosystems to exploit the novel habitat or food resources and this ecological shift between the two populations could have been the driving force for genetic divergence and speciation [78]. Due to the considerable lack of ecological data on *L. neilli*, we can only propose some hypotheses on this point but we suggest that this ecological shift could affect the reproductive behaviour of these rodents and we suspect *L. neilli* to use caves for breeding and nursing as young individuals and babies have been observed within caves during our survey. The ecological specialization of *L. neilli* to limestone karsts could also be associated to the particular xerophytic flora of karsts or to specific food resources available in caves such as insects.

However, an allopatric speciation of *L. neilli* and *L. edwardsi* followed by a more recent sympatry cannot be excluded. The two species could have evolved independently in different regions of Southeast Asia during Pliocene and subsequently dispersed throughout Indochina to establish the current parapatry in northern Thailand. Additional data about the past (and present) distribution of *L. neilli*, *L. edwardsi* and their ancestor are needed to infer the speciation process leading to the origin of a cave-dwelling rodent.

The relative role of vicariance (the fragmentation of populations due to isolating barriers) and dispersal (the colonization of new habitats across potential pre-existing barriers) to explain biogeographic patterns of cave and karstic endemic taxa have also been widely discussed during decades and the debate is still ongoing [1], [2]. The first step to differentiate these two hypotheses is to identify potential dispersal barriers. Three main barriers associated with the Central Plain of Thailand have been detected within the studied area by VIP (Figure 7). The Central Plain of Thailand, a rift valley 500 km long and up to 200 km wide [79], [80], is the result of the tectonic deformation of this region due to the collision of the Indian and Eurasian continental plates throughout the Cenozoic Era. As a consequence, Quaternary sediments have been accumulated on the valley floor and progressively buried the basement rocks of the Central Plain [79], [81]. These deposits, with thickness of about 2000 m, represent a complex sequence of alluvial, fluvial, and deltaic sediments and were overlaid in the Lower Central Plain with thick marine clay during Holocene sea transgression [82].

The role of these barriers in the evolutionary history of *L. neilli* has been tested using ancestral range reconstruction analysis and ABC methods and they clearly supported the vicariant hypothesis. All four of the current groups of *L. neilli* originate from a common ancestral population widely distributed within Thailand during Pliocene and whose effective population size was several times higher than those of the current groups. Then the formation of the Central Plain of Thailand during Quaternary has played a significant role in progressively fragmenting the ancestral population and shaping the phylogeographic pattern of this species. The progressive burying and disappearing of limestone karsts of the Central Plain and of the main river valley basins would constitute a barrier to *L. neilli* dispersal and lead to the vicariant events isolating populations. The western populations were the first to have been isolated around 2.4 Myr, followed by the central populations around 1.3 Myr and finally the northern and northeastern groups around 0.9 Myr.

The non significant Mantel's tests performed on both mitochondrial and nuclear datasets supported the hypothesis of the presence of isolating barriers within this region. Geographic distance alone does not seem to influence the genetic structure of *L. neilli* and even short distances of lowland areas among karsts seem to constitute important dispersal barriers. The high *F*_ST_ values for both mitochondrial and nuclear datasets between western and central populations (Table 4) highlighted the lack of gene flow between these two regions and the influence of the formation of the Central Plain of Thailand on *L. neilli* dispersal.

### Implication for the conservation of *L. neilli* and limestone karsts

*L. neilli* is currently classified as “Data deficient” on the IUCN Red List due to the lack of data about its distribution and ecological requirements [23], but our study provides valuable information for the re-evaluation of its status. Our survey of the rodent diversity in Thai limestone karsts has shown that the distribution range of this species is wider and its populations more numerous than indicated by previous records. Therefore, we believe that *L. neilli* does not satisfy the criteria of the IUCN threatened categories now but is likely to qualify for a threatened category in the near future for the following reasons.

First, populations of *L. neilli* are small and highly fragmented. Six Evolutionarily Significant Units (ESUs) have been defined in Latinne *et al.* [25] on the basis of mtDNA diversity. However, the larger sampling of the present study and additional information obtained from nuclear genes indicate that the delineation of four ESUs is more appropriate. The western, central, northern and northeastern groups of populations were historically isolated and evolved independently. Consequently, their preservation is of major importance to protect the intraspecific diversity of this species [83].

Second, the habitat requirement of *L. neilli* seems to be highly specific and the species is closely associated with limestone karsts. This habitat is currently strongly threatened through quarrying, hunting, and urbanization [13], [14]. More than 122 limestone quarries are operating in Thailand in all the provinces where limestone is available [84]. More than 20% of limestone karsts in Thailand have already been quarried for cement, lime and hard core, and many have completely disappeared from the landscape [85]. As a result of these activities, some karst endemic species became dramatically endangered. The long-term survival of *L. neilli* is thus strongly dependent on the preservation of karstic habitats.

Third, *L. neilli* is intensively trapped for human consumption in some regions of northeastern Thailand. This could threaten the long-term preservation of the species in this region.

Therefore, we strongly recommend classifying this species at least as “Near Threatened” on the IUCN Red List. If the four ESUs that we described were assessed separately, they would qualify as “Vulnerable” because of their small geographic ranges and population sizes. Particular attention should be paid to the western lineages, highly divergent for both mitochondrial (more than 7% of net K2P distance for cytb) and nuclear genes, which could be considered as a distinct sub-species of *L. neilli* under the phylogenetic species concept [25] and therefore requires separate management and conservation plans.

We also suggest, following the recommendations of Vermeulen & Whitten [14], for limestone quarrying management, and specially to avoid the quarrying of isolated limestone hills and to locate quarries in the largest limestone areas and let a considerable part of it remain intact.

## Conclusions

This study reveals a complex genetic structure for *Leopoldamys neilli* supported by both mitochondrial and nuclear markers. The evolutionary history of this species has been influenced by the geological history of Thailand during Plio-Pleistocene and vicariance has played a significant role in shaping the phylogeographic pattern of *L. neilli*. The western, central, northern and northeastern groups of populations were historically isolated and they evolved independently. To our knowledge this is the first phylogeographic study of karst endemic taxa in this region. Further phylogeographic studies of other co-distributed taxa will allow the determination of whether the phylogeographic pattern recovered for *L. neilli* and the strong isolation of western populations could be generalized to other species living in Thai limestone karsts and whether they share a similar evolutionary history.

## Supporting Information

Figure S1 **Map of localities sampled during our survey of rodent diversity in Thai limestone karsts.**
(TIF)

Figure S2 **Results** **of the Bayesian clustering analysis with STRUCTURE.** (A) Plot of the likelihood of the mean Ln Pr (X|K). (B) Values of *ΔK* calculated according to Evanno *et al.* (2005).
(TIF)

Figure S3 **Comparison of the posterior probabilities of all scenarios for each of four steps of our ABC analysis.**
(TIF)

Table S1 **Sampling locality, samples used for sequencing for each dataset (n) and haplotype/allele distribution.**
(DOC)

Table S2 **GenBank Accession numbers for cytb, COI, bfibr and G6pd haplotypes/alleles of** ***L. neilli*** **and outgroup sequences (** ***L. edwardsi*** **and** ***L. sabanus*** **).**
(DOC)

Table S3 **Primers and PCR conditions used in this study.**
(DOC)

Table S4 **Nuclear allele frequency among the main lineages of** ***L. neilli*** **.**
(DOC)

Table S5 **Pairwise genetic divergence (net K2P distance) for the cytb dataset among lineages of** ***L. neilli*** **.**
(DOC)

Table S6 **Pairwise** ***F*** **_ST_ values based on microsatellite dataset among clusters (Geneland analysis) containing more than three individuals.**
(DOC)

Table S7 **Estimations of the posterior distribution of the effective population sizes for the best scenario of each step revealed by the ABC analysis.**
(DOC)
