# Supplementary figures and images for: Combined Mitochondrial and Nuclear Markers Revealed a Deep Vicariant History for Leopoldamys neilli, a Cave-Dwelling Rodent of Thailand
Source: PLoS One. 2012 Oct 31;7(10):e47670. doi: 10.1371/journal.pone.0047670 (PMC3485250; doi:10.1371/journal.pone.0047670)

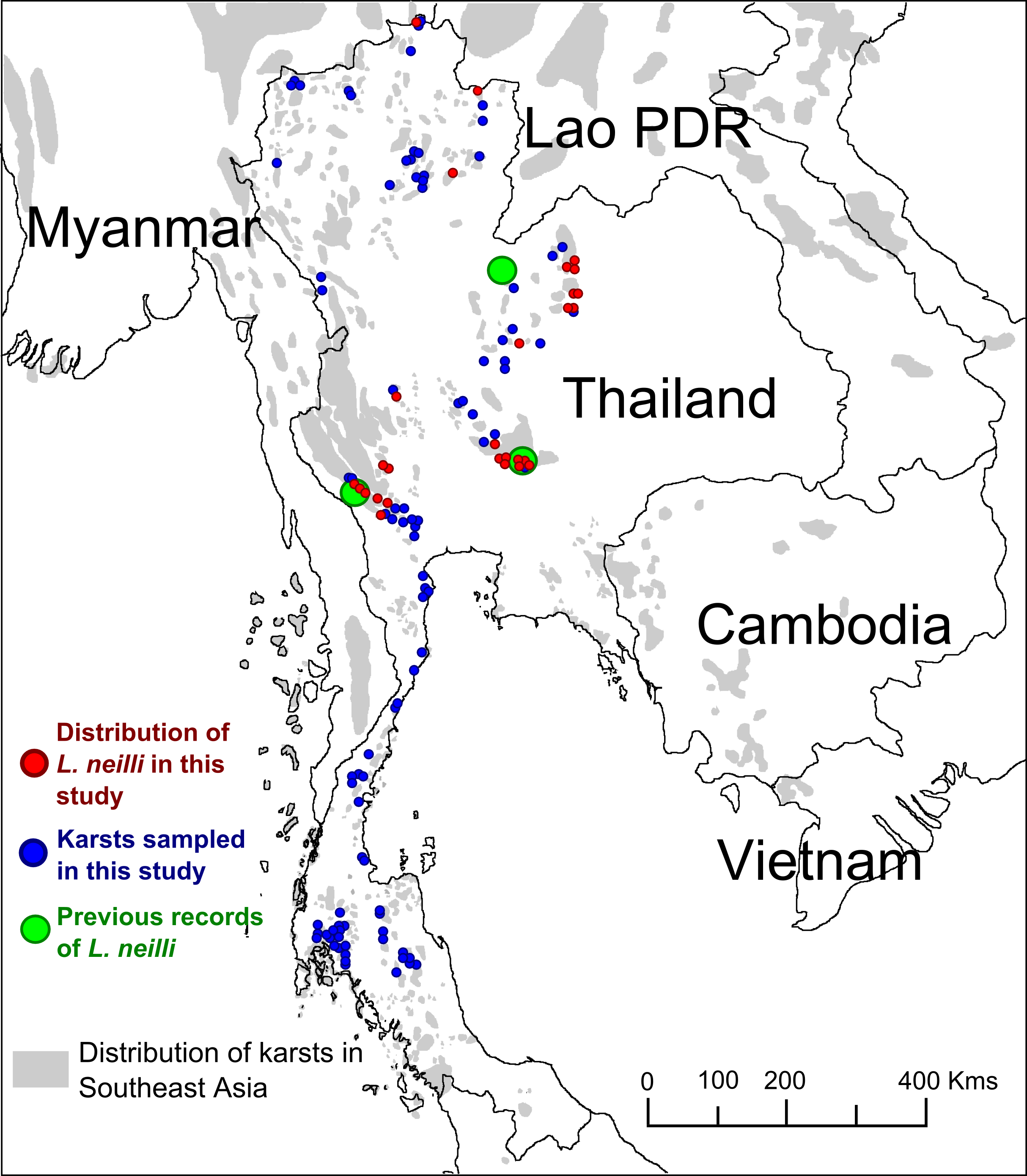

Supplement: Figure S1 — Map of localities sampled during our survey of rodent diversity in Thai limestone karsts. (TIF) [file pone.0047670.s001.tif]

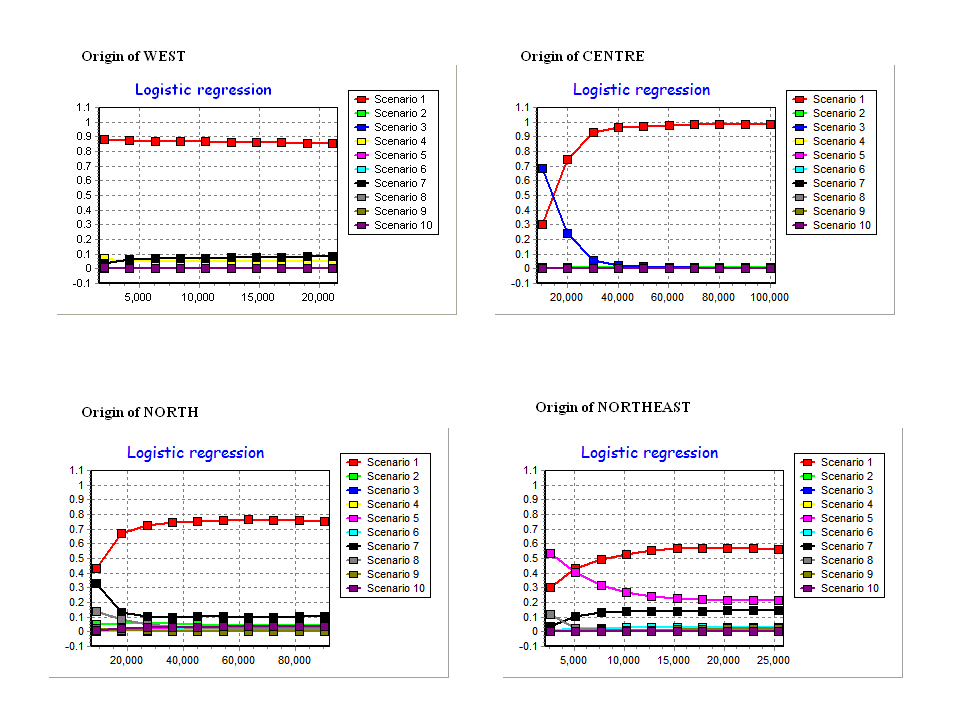

Supplement: Figure S3 — Comparison of the posterior probabilities of all scenarios for each of four steps of our ABC analysis. (TIF) [file pone.0047670.s003.tif]
