# Supplementary material for: Combined Mitochondrial and Nuclear Markers Revealed a Deep Vicariant History for Leopoldamys neilli, a Cave-Dwelling Rodent of Thailand
Source: PLoS One. 2012 Oct 31;7(10):e47670. doi: 10.1371/journal.pone.0047670 (PMC3485250; doi:10.1371/journal.pone.0047670)
Supplement: Table S1 — Sampling locality, samples used for sequencing for each dataset (n) and haplotype/allele distribution. (DOC) [file pone.0047670.s004.doc]

| **Locality ID** | **Province** | **Coord (Lat-Long)** | **n**  **mt**  **genes** | **n**  **nu genes** | **n**  **microsat** | **cytb haplotypes** | **COI haplotypes** | **bfibr**  **alleles** | **G6PD**  **alleles** |
| --- | --- | --- | --- | --- | --- | --- | --- | --- | --- |
| KAN1 | Kanchanaburi | 14°14’  99°03’ | 1 | 1 | 1 | HCB16 (1) | HCOI14 (1) | HBF31 (2) | HG6PD5 (2) |
| KAN2 | Kanchanaburi | 14°12’  99°07’ | 12 | 5 | 12 | HCB17 (11)  HCB20 (1) | HCOI14 (1)  HCOI15 (11) | HBF29 (2)  HBF30 (2)  HBF31 (5)  HBF32 (1) | HG6PD5 (8)  HG6PD6 (2) |
| KAN3 | Kanchanaburi | 14°12’  99°08’ | 2 | 2 | 2 | HCB16 (1)  HCB29 (1) | HCOI14 (1)  HCOI16 (1) | HBF29 (1)  HBF30 (1)  HBF31 (2) | HG6PD5 (4) |
| KAN4 | Kanchanaburi | 14°08’  99°17’ | 15 | 4 | 15 | HCB16 (14)  HCB20 (1) | HCOI12 (3)  HCOI14 (12) | HBF31 (8) | HG6PD5 (8) |
| KAN5 | Kanchanaburi | 14°03’  99°25’ | 7 | 4 | 7 | HCB16 (4)  HCB18 (2)  HCB19 (1) | HCOI12 (2)  HCOI14 (4)  HCOI18 (1) | HBF30 (2)  HBF31 (5)  HBF33 (1) | HG6PD5 (8) |
| KAN6 | Kanchanaburi | 13°54’  99°18’ | 3 | 2 | 3 | HCB16 (2)  HCB17 (1) | HCOI12 (2)  HCOI13 (1) | HBF31 (3)  HBF33 (1) | HG6PD5 (4) |
| KAN7 | Kanchanaburi | 14°30’  99°24’ | 16 | 5 | 16 | HCB21 (4)  HCB22 (12) | HCOI14 (16) | HBF29 (4)  HBF31 (3)  HBF34 (1)  HBF38 (2) | HG6PD5 (6)  HG6PD6 (4) |
| KAN8 | Kanchanaburi | 14°28’  99°25’ | 6 | 5 | 6 | HCB21 (3)  HCB22 (3) | HCOI14 (6) | HBF14 (2)  HBF31 (5)  HBF35 (2)  HBF36 (1) | HG6PD5 (5)  HG6PD6 (5) |
| UT | Uthai Thani | 15°29'  99°34' | 8 | 6 | 8 | HCB34 (1)  HCB35 (5)  HCB36 (1)  HCB37 (1) | HCOI25 (8) | HBF14 (7)  HBF6 (3)  HBF28 (2) | HG6PD5 (12) |
| LOP1 | Lopburi | 14°48’  100°49’ | 5 | 4 | 5 | HCB15 (3)  HCB27 (1)  HCB28 (1) | HCOI11 (4)  HCOI17 (1) | HBF6 (1)  HBF9 (3)  HBF10 (2)  HBF37 (2) | HG6PD4 (8) |
| SARA1 | Saraburi | 14°39’  100°58’ | 8 | 4 | 8 | HCB13 (4)  HCB14 (2)  HCB26 (2) | HCOI9 (6)  HCOI11(2) | HBF9 (7)  HBF10 (1) | HG6PD4 (8) |
| SARA2 | Saraburi | 14°40’  101°00’ | 2 | 1 | 2 | HCB13 (2) | HCOI10 (2) | HBF10 (2) | HG6PD4 (2) |
| SARA3 | Saraburi | 14°38’  101°08’ | 1 | 1 | 1 | HCB10 (1) | HCOI8 (1) | HBF9 (2) | HG6PD4 (2) |
| SARA4 | Saraburi | 14°34’  101°08’ | 15 | 5 | 15 | HCB10 (7)  HCB11 (4)  HCB12 (1)  HCB13 (2)  HCB25 (1) | HCOI8 (12)  HCOI11(3) | HBF9 (7)  HBF10 (3) | HG6PD4 (10) |
| SARA5 | Saraburi | 14°39'  100°58' | 5 | 3 | 5 | HCB10 (1)  HCB13 (2)  HCB14 (2) | HCOI8 (1)  HCOI9 (2)  HCOI11 (2) | HBF9 (4)  HBF10 (2) | HG6PD4 (6) |
| NKR1 | Nakhon Ratchasima | 14°35’  101°14’ | 1 | 1 | 1 | HCB10 (1) | HCOI8 (1) | HBF9 (2) | HG6PD4 (2) |
| NKR2 | Nakhon Ratchasima | 14°33’  101°18’ | 14 | 5 | 14 | HCB10 (3)  HCB11 (11) | HCOI8 (14) | HBF9 (10) | HG6PD4 (10) |
| PET | Petchabun | 16°14'  101°08' | 3 | 3 | 3 | HCB32 (3) | HCOI23 (3) | HBF4 (3)  HBF20 (3) | HG6PD2 (2)  HG6PD7 (4) |
| CHAI1 | Chaiyaphum | 16°34'  101°52' | 6 | 6 | 6 | HCB30 (6) | HCOI19 (6) | HBF4 (6)  HBF19 (5)  HBF25 (1) | HG6PD2 (12) |
| CHAI2 | Chaiyaphum | 16°34'  101°51' | 1 | 1 | 1 | HCB31 (1) | HCOI20 (1) | HBF4 (1)  HBF25 (1) | HG6PD2 (2) |
| KK1 | Khon Kaen | 16°43'  101°56' | 10 | 5 | 10 | HCB2 (2)  HCB3 (8) | HCOI1 (3)  HCOI21(7) | HBF4 (8)  HBF25 (2) | HG6PD1 (10) |
| KK2 | Khon Kaen | 16°44'  101°55' | 15 | 5 | 15 | HCB3 (15) | HCOI1 (13)  HCOI22(2) | HBF4 (6)  HBF19 (4) | HG6PD1 (4)  HG6PD7 (6) |
| LO1 | Loei | 17° 05'  101°47' | 17 | 4 | 17 | HCB3 (5)  HCB4 (5)  HCB5 (6)  HCB6 (1) | HCOI1 (14)  HCOI2 (1)  HCOI3 (2) | HBF1 (1)  HBF4 (5)  HBF17 (2) | HG6PD1 (6)  HG6PD2 (2) |
| LO2 | Loei | 17° 03'  101°54' | 14 | 4 | 14 | HCB1 (7)  HCB2 (6)  HCB3 (1) | HCOI1 (13)  HCOI4 (1) | HBF2 (4)  HBF4 (3)  HBF20 (1) | HG6PD1 (8) |
| LO3 | Loei | 17° 06'  101°56' | 9 | 4 | 9 | HCB1 (7)  HCB2 (2) | HCOI1(9) | HBF2 (2)  HBF3 (3)  HBF17 (1)  HBF18 (1)  HBF19 (1) | HG6PD1 (8) |
| PHR | Phrae | 18°22’  100°21’ | 11 | 6 | 11 | HCB9 (11) | HCOI7 (11) | HBF6 (7)  HBF8 (2)  HBF19 (1)  HBF24 (2) | HG6PD3 (12) |
| NAN | Nan | 19°23’  100°36’ | 16 | 6 | 16 | HCB7 (3)  HCB8 (11)  HCB23 (1)  HCB24 (1) | HCOI5 (3)  HCOI6 (13) | HBF4 (2)  HBF5 (4)  HBF19 (1)  HBF21 (1)  HBF22 (3)  HBF23 (1) | HG6PD3 (12) |
| CHR | Chiang Rai | 20°20'  99°51' | 2 | 2 | 2 | HCB33 (2) | HCOI24 (2) | HBF4 (1)  HBF6 (1)  HBF26 (1)  HBF27 (1) | HG6PD7 (1)  HG6PD8 (3) |
