# Supplementary material for: Combined Mitochondrial and Nuclear Markers Revealed a Deep Vicariant History for Leopoldamys neilli, a Cave-Dwelling Rodent of Thailand
Source: PLoS One. 2012 Oct 31;7(10):e47670. doi: 10.1371/journal.pone.0047670 (PMC3485250; doi:10.1371/journal.pone.0047670)
Supplement: Table S2 — GenBank Accession numbers for cytb, COI, bfibr and G6pd haplotypes/alleles of L. neilli and outgroup sequences ( L. edwardsi and L. sabanus ). (DOC) [file pone.0047670.s005.doc]

| **cytb** | | **COI** | | **bfibr** | | **G6PD** | |
| --- | --- | --- | --- | --- | --- | --- | --- |
| Haplotype | Accession no. | Haplotype | Accession no. | Allele | Accession no. | Allele | Accession no. |
| ***Leopoldamys neilli*** | | | | | | | |
| HCB1 | HM219591 | HCOI1 | HM219573 | HBF2 | HM219556 | HG6PD1 | JQ081343 |
| HCB2 | HM219592 | HCOI2 | HM219574 | HBF3 | HM219557 | HG6PD2 | JQ081344 |
| HCB3 | HM219593 | HCOI3 | HM219575 | HBF4 | HM219558 | HG6PD3 | JQ081345 |
| HCB4 | HM219594 | HCOI4 | HM219576 | HBF5 | HM219559 | HG6PD4 | JQ081346 |
| HCB5 | HM219595 | HCOI5 | HM219577 | HBF6 | HM219560 | HG6PD5 | JQ081347 |
| HCB6 | HM219596 | HCOI6 | HM219578 | HBF8 | HM219562 | HG6PD6 | JQ081348 |
| HCB7 | HM219597 | HCOI7 | HM219579 | HBF9 | HM219563 | HG6PD7 | JQ081349 |
| HCB8 | HM219598 | HCOI8 | HM219580 | HBF10 | HM219564 | HG6PD8 | JQ081350 |
| HCB9 | HM219599 | HCOI9 | HM219581 | HBF14 | HM219568 |  |  |
| HCB10 | HM219600 | HCOI10 | HM219582 | HBF17 | JQ081320 |  |  |
| HCB11 | HM219601 | HCOI11 | HM219583 | HBF18 | JQ081321 |  |  |
| HCB12 | HM219602 | HCOI12 | HM219584 | HBF19 | JQ081322 |  |  |
| HCB13 | HM219603 | HCOI13 | HM219585 | HBF20 | JQ081323 |  |  |
| HCB14 | HM219604 | HCOI14 | HM219586 | HBF21 | JQ081324 |  |  |
| HCB15 | HM219605 | HCOI15 | HM219587 | HBF22 | JQ081325 |  |  |
| HCB16 | HM219606 | HCOI16 | HM219588 | HBF23 | JQ081326 |  |  |
| HCB17 | HM219607 | HCOI17 | JQ081310 | HBF24 | JQ081327 |  |  |
| HCB18 | HM219608 | HCOI18 | JQ081311 | HBF25 | JQ081328 |  |  |
| HCB19 | HM219609 | HCOI19 | JQ081312 | HBF26 | JQ081329 |  |  |
| HCB20 | HM219610 | HCOI20 | JQ081313 | HBF27 | JQ081330 |  |  |
| HCB21 | HM219611 | HCOI21 | JQ081314 | HBF28 | JQ081331 |  |  |
| HCB22 | HM219612 | HCOI22 | JQ081315 | HBF29 | JQ081332 |  |  |
| HCB23 | JQ081356 | HCOI23 | JQ081316 | HBF30 | JQ081333 |  |  |
| HCB24 | JQ081357 | HCOI24 | JQ081317 | HBF31 | JQ081334 |  |  |
| HCB25 | JQ081358 | HCOI25 | JQ081318 | HBF32 | JQ081335 |  |  |
| HCB26 | JQ081359 |  |  | HBF33 | JQ081336 |  |  |
| HCB27 | JQ081360 |  |  | HBF34 | JQ081337 |  |  |
| HCB28 | JQ081361 |  |  | HBF35 | JQ081338 |  |  |
| HCB29 | JQ081362 |  |  | HBF36 | JQ081339 |  |  |
| HCB30 | JQ081363 |  |  | HBF37 | JQ081340 |  |  |
| HCB31 | JQ081364 |  |  | HBF38 | JQ081341 |  |  |
| HCB32 | JQ081365 |  |  |  |  |  |  |
| HCB33 | JQ081366 |  |  |  |  |  |  |
| HCB34 | JQ081367 |  |  |  |  |  |  |
| HCB35 | JQ081368 |  |  |  |  |  |  |
| HCB36 | JQ081369 |  |  |  |  |  |  |
| HCB37 | JQ081370 |  |  |  |  |  |  |
| **Outgroup: *Leopoldamys edwardsi*** | | | | | | | |
| R4222 | HM217444 | R4222 | HM217571 | R4222 | HQ454297 | R4222 | JQ081352 |
| R4296 | HM217450 | R4296 | HM217577 | R4296 | HQ454292 | R4296 | JQ081353 |
| R4370 | HM217451 | R4370 | HM217578 | R4370 | HQ454293 | R4370 | JQ081351 |
| **Outgroup: *Leopoldamys sabanus*** | | | | | | | |
| R3033 | HM217400 | R3033 | HM217531 | R3033 | HQ454288 | R3033 | JQ081354 |
| R3111 | HM217404 | R3111 | HM217534 | R3111 | - | R3111 | - |
| L288 | JQ081371 | L288 | JQ081319 | L288 | JQ081342 | L288 | JQ081355 |
