# Supplementary material for: Combined Mitochondrial and Nuclear Markers Revealed a Deep Vicariant History for Leopoldamys neilli, a Cave-Dwelling Rodent of Thailand
Source: PLoS One. 2012 Oct 31;7(10):e47670. doi: 10.1371/journal.pone.0047670 (PMC3485250; doi:10.1371/journal.pone.0047670)
Supplement: Table S3 — Primers and PCR conditions used in this study. (DOC) [file pone.0047670.s006.doc]

| **Marker** | **Primer name** | **Primer sequence** | **Annealing**  **temperature** | **Reference** |
| --- | --- | --- | --- | --- |
| **cytb** | LneilliFw | 5’-TCCATCCAACATCTCATCATG-3’ | 50°C | Latinne *et al.* 2011 |
|  | LneilliRv | 5’-GGAGGCTAGTTGGCCAATG-3’ |  |  |
| **COI** | BatL5310 | 5’-CCTACTCRGCCATTTTACCTATG-3’ | 50°C | Robins *et al.* 2007 |
|  | R6036R | 5’-ACTTCTGGGTGTCCAAAGAATCA-3’ |  |  |
| **bfibr** | BFIBR1 | 5’-ATTCACAACGGCATGTTCTTCAG-3’ | 58.7°C | Seddon *et al.* 2001 |
|  | BFIBR2 | 5’-AANGKCCACCCCAGTAGTATCTG-3’ |  |  |
| **G6pd** | G6pd-int1L | 5’-CAGATCTGTGAACGTGTTTGG-3’ | 56.5°C | Iwasa & Suzuki 2002 |
|  | G6pd-int1H | 5’-GGTACAACTCTTCCCTCAGG-3’ |  |  |

**References:**

Iwasa MA, Suzuki H (2002) Evolutionary significance of chromosome changes in northeastern Asiatic red-backed voles inferred with the aid of intron 1 sequences of the G6pd gene. Chromosome Res 10: 419-428.

Latinne A, Waengsothorn S, Herbreteau V, Michaux J (2011) Evidence of complex phylogeographic structure for the threatened rodent *Leopoldamys neilli*, in Southeast Asia. Conserv Genet 12: 1495-1511.

Robins JH, Hingston M, Matisoo-Smith E, Ross HA (2007) Identifying *Rattus* species using mitochondrial DNA. Mol Ecol Notes 7: 717-729.

Seddon JM, Santucci F, Reeve NJ, Hewitt GM (2001) DNA footprints of European hedgehogs, *Erinaceus europaeus* and *E. concolor*. Pleistocene refugia, postglacial expansion and colonization routes. Mol Ecol 10: 2187-2198.
