# Supplementary material for: Combined Mitochondrial and Nuclear Markers Revealed a Deep Vicariant History for Leopoldamys neilli, a Cave-Dwelling Rodent of Thailand
Source: PLoS One. 2012 Oct 31;7(10):e47670. doi: 10.1371/journal.pone.0047670 (PMC3485250; doi:10.1371/journal.pone.0047670)
Supplement: Table S4 — Nuclear allele frequency among the main lineages of L. neilli . (DOC) [file pone.0047670.s007.doc]

| **Marker** | **Allele** | **Frequency** | | | | | | | | | |
| --- | --- | --- | --- | --- | --- | --- | --- | --- | --- | --- | --- |
|  |  | Loei/KK | Petchabun | Chaiyaphum | Chiang Rai | Nan | Phrae | Centre1 | Centre2 | Uthai Thani | Kanchanaburi |
| **bfibr** | HBF37 |  |  |  |  |  |  | 0.0833 |  |  |  |
|  | HBF9 |  |  |  |  |  |  | 0.5417 | 0.9167 |  |  |
|  | HBF10 |  |  |  |  |  |  | 0.3333 | 0.0833 |  |  |
|  | HBF3 | 0.0682 |  |  |  |  |  |  |  |  |  |
|  | HBF18 | 0.0227 |  |  |  |  |  |  |  |  |  |
|  | HBF17 | 0.0682 |  |  |  |  |  |  |  |  |  |
|  | HBF2 | 0.1591 |  |  |  |  |  |  |  |  |  |
|  | HBF20 | 0.0227 | 0.5000 |  |  |  |  |  |  |  |  |
|  | HBF25 | 0.0455 |  | 0.1429 |  |  |  |  |  |  |  |
|  | HBF4 | 0.5000 | 0.5000 | 0.5000 | 0.2500 | 0.1667 |  |  |  |  |  |
|  | HBF26 |  |  |  | 0.2500 |  |  |  |  |  |  |
|  | HBF27 |  |  |  | 0.2500 |  |  |  |  |  |  |
|  | HBF22 |  |  |  |  | 0.2500 |  |  |  |  |  |
|  | HBF23 |  |  |  |  | 0.0833 |  |  |  |  |  |
|  | HBF5 |  |  |  |  | 0.3333 |  |  |  |  |  |
|  | HBF21 |  |  |  |  | 0.0833 |  |  |  |  |  |
|  | HBF24 |  |  |  |  |  | 0.1667 |  |  |  |  |
|  | HBF8 |  |  |  |  |  | 0.1667 |  |  |  |  |
|  | HBF19 | 0.1136 |  | 0.3571 |  | 0.0833 | 0.0833 |  |  |  |  |
|  | HBF6 |  |  |  | 0.2500 |  | 0.5833 | 0.0417 |  | 0.2500 |  |
|  | HBF28 |  |  |  |  |  |  |  |  | 0.1667 |  |
|  | HBF14 |  |  |  |  |  |  |  |  | 0.5833 | 0.0357 |
|  | HBF29 |  |  |  |  |  |  |  |  |  | 0.1250 |
|  | HBF34 |  |  |  |  |  |  |  |  |  | 0.0179 |
|  | HBF38 |  |  |  |  |  |  |  |  |  | 0.0357 |
|  | HBF36 |  |  |  |  |  |  |  |  |  | 0.0179 |
|  | HBF35 |  |  |  |  |  |  |  |  |  | 0.0357 |
|  | HBF32 |  |  |  |  |  |  |  |  |  | 0.0179 |
|  | HBF31 |  |  |  |  |  |  |  |  |  | 0.5893 |
|  | HBF33 |  |  |  |  |  |  |  |  |  | 0.0357 |
|  | HBF30 |  |  |  |  |  |  |  |  |  | 0.0893 |
| **G6pd** | HG6PD5 |  |  |  |  |  |  |  |  | 1.0000 | 0.8036 |
|  | HG6PD6 |  |  |  |  |  |  |  |  |  | 0.1964 |
|  | HG6PD3 |  |  |  |  | 1.0000 | 1.0000 |  |  |  |  |
|  | HG6PD2 | 0.0455 | 0.3333 | 1.0000 |  |  |  |  |  |  |  |
|  | HG6PD7 | 0.1364 | 0.6667 |  | 0.2500 |  |  |  |  |  |  |
|  | HG6PD1 | 0.8182 |  |  |  |  |  |  |  |  |  |
|  | HG6PD8 |  |  |  | 0.7500 |  |  |  |  |  |  |
|  | HG6PD4 |  |  |  |  |  |  | 1.0000 | 1.0000 |  |  |
