# Supplementary material for: Combined Mitochondrial and Nuclear Markers Revealed a Deep Vicariant History for Leopoldamys neilli, a Cave-Dwelling Rodent of Thailand
Source: PLoS One. 2012 Oct 31;7(10):e47670. doi: 10.1371/journal.pone.0047670 (PMC3485250; doi:10.1371/journal.pone.0047670)
Supplement: Table S5 — Pairwise genetic divergence (net K2P distance) for the cytb dataset among lineages of L. neilli . (DOC) [file pone.0047670.s008.doc]

|  | Loei/KK | Petchabun | Chaiyaphum | Chiang Rai | Nan | Phrae | Centre1 | Centre2 | Uthai Thani |
| --- | --- | --- | --- | --- | --- | --- | --- | --- | --- |
| Loei/KK |  |  |  |  |  |  |  |  |  |
| Petchabun | 0.005 |  |  |  |  |  |  |  |  |
| Chaiyaphum | 0.007 | 0.012 |  |  |  |  |  |  |  |
| Chiang Rai | 0.012 | 0.016 | 0.014 |  |  |  |  |  |  |
| Nan | 0.015 | 0.021 | 0.023 | 0.023 |  |  |  |  |  |
| Phrae | 0.007 | 0.012 | 0.014 | 0.014 | 0.018 |  |  |  |  |
| Centre1 | 0.028 | 0.029 | 0.031 | 0.035 | 0.041 | 0.031 |  |  |  |
| Centre2 | 0.030 | 0.031 | 0.038 | 0.038 | 0.043 | 0.034 | 0.026 |  |  |
| Uthai Thani | 0.074 | 0.080 | 0.075 | 0.074 | 0.081 | 0.074 | 0.080 | 0.088 |  |
| Kanchanaburi | 0.075 | 0.079 | 0.074 | 0.081 | 0.074 | 0.082 | 0.079 | 0.087 | 0.013 |
