# Supplementary material for: Combined Mitochondrial and Nuclear Markers Revealed a Deep Vicariant History for Leopoldamys neilli, a Cave-Dwelling Rodent of Thailand
Source: PLoS One. 2012 Oct 31;7(10):e47670. doi: 10.1371/journal.pone.0047670 (PMC3485250; doi:10.1371/journal.pone.0047670)
Supplement: Table S6 — Pairwise F ST values based on microsatellite dataset among clusters (Geneland analysis) containing more than three individuals. (DOC) [file pone.0047670.s009.doc]

|  | **Cl.2** | **Cl.3** | **Cl.4** | **Cl.5** | **Cl.6** | **Cl.7** | **Cl.8** | **Cl.9** | **Cl.10** | **Cl.11** | **Cl.12** |
| --- | --- | --- | --- | --- | --- | --- | --- | --- | --- | --- | --- |
| **Cl.1** (LO2-3) | 0.097 | 0.212 | 0.099 | 0.246 | 0.216 | 0.299 | 0.161 | 0.209 | 0.235 | 0.158 | 0.324 |
| **Cl.2** (LO1) |  | 0.214 | 0.103 | 0.180 | 0.144 | 0.261 | 0.148 | 0.186 | 0.199 | 0.133 | 0.324 |
| **Cl.3** (CHAI1) |  |  | 0.214 | 0.341 | 0.254 | 0.389 | 0.237 | 0.308 | 0.319 | 0.204 | 0.444 |
| **Cl.4** (CHAI2 +KK1-2+PET) |  |  |  | 0.229 | 0.194 | 0.284 | 0.159 | 0.203 | 0.245 | 0.165 | 0.307 |
| **Cl.5** (UT) |  |  |  |  | 0.229 | 0.310 | 0.287 | 0.312 | 0.269 | 0.214 | 0.426 |
| **Cl.6** (NAN) |  |  |  |  |  | 0.320 | 0.207 | 0.250 | 0.233 | 0.150 | 0.345 |
| **Cl.7** (PHR) |  |  |  |  |  |  | 0.310 | 0.350 | 0.340 | 0.272 | 0.491 |
| **Cl.8** (SARA3-4+NKR1-2) |  |  |  |  |  |  |  | 0.133 | 0.254 | 0.193 | 0.345 |
| **Cl.9** (SARA1-2-5+LOP1) |  |  |  |  |  |  |  |  | 0.257 | 0.180 | 0.400 |
| **Cl.10** (KAN1-2) |  |  |  |  |  |  |  |  |  | 0.117 | 0.331 |
| **Cl.11** (KAN3-4-5-6) |  |  |  |  |  |  |  |  |  |  | 0.235 |
| **Cl.12** (KAN7-8) |  |  |  |  |  |  |  |  |  |  |  |
