# Supplementary material for: Combined Mitochondrial and Nuclear Markers Revealed a Deep Vicariant History for Leopoldamys neilli, a Cave-Dwelling Rodent of Thailand
Source: PLoS One. 2012 Oct 31;7(10):e47670. doi: 10.1371/journal.pone.0047670 (PMC3485250; doi:10.1371/journal.pone.0047670)
Supplement: Table S7 — Estimations of the posterior distribution of the effective population sizes for the best scenario of each step revealed by the ABC analysis. (DOC) [file pone.0047670.s010.doc]

| Step | Best scenario | Pop | Mean effective population size | Median | Mode | 95% CI |
| --- | --- | --- | --- | --- | --- | --- |
| **Origin of WEST (W)** | W.1 Only from AP | AP | 34000 | 33200 | 30200 | 6780-49700 |
|  |  | W | 22900 | 21700 | 17500 | 4320-46800 |
|  |  | C | 13500 | 10500 | 6400 | 1680-42200 |
|  |  | N | 6680 | 3940 | 1760 | 406-33000 |
|  |  | NE | 13700 | 10800 | 7580 | 1390-42900 |
| **Origin of CENTRE (C)** | C.1 Only from AP | AP | 39600 | 41300 | 45400 | 10720-51600 |
|  |  | W | 25000 | 24600 | 24200 | 5270-48000 |
|  |  | C | 14000 | 11100 | 5630 | 1290-41500 |
|  |  | N | 10800 | 7810 | 4210 | 979-39600 |
|  |  | NE | 24100 | 23400 | 16900 | 4360-47300 |
| **Origin of NORTH (N**) | N.1 Only from AP | AP | 35100 | 35400 | 34100 | 9800-50500 |
|  |  | W | 17600 | 15600 | 12000 | 2630-44500 |
|  |  | C | 12800 | 9950 | 7270 | 1400-41800 |
|  |  | N | 6360 | 3640 | 1460 | 367-33200 |
|  |  | NE | 14600 | 11600 | 8220 | 1970-43800 |
| **Origin of NORTHEAST (NE)** | NE.1 Only from AP | AP | 35100 | 35600 | 36800 | 10500-50700 |
|  |  | W | 20000 | 18200 | 14100 | 3220-46100 |
|  |  | C | 13600 | 10700 | 8010 | 1390-42400 |
|  |  | N | 6990 | 4180 | 2840 | 442-34800 |
|  |  | NE | 17800 | 15300 | 11800 | 2330-45500 |
